# Engineered chimeric insecticidal crystalline protein improves resistance to lepidopteran insects in rice (*Oryza sativa L.*) and maize (*Zea mays L.*)

**DOI:** 10.1038/s41598-022-16426-6

**Published:** 2022-07-22

**Authors:** Yang Liu, Siping Han, Shuo Yang, Ziqi Chen, Yuejia Yin, Jinghui Xi, Qing Liu, Weiyu Yan, Xinyuan Song, Fangfang Zhao, Jia Guo, Xiangguo Liu, Dongyun Hao

**Affiliations:** 1 Institute of Agricultural Biotechnology, Jilin Academy of Agricultural Sciences, Changchun, China; 2 College of Life Science, Jilin University, ChangChun, China; 3 College of Plant Science, Jilin University, ChangChun, China; 4 College of Life Science and Technology, Harbin Normal University, Harbin, China

**Keywords:** Biotechnology, Plant biotechnology, Agricultural genetics, Molecular engineering in plants

## Abstract

The insecticidal crystalline proteins (Crys) are a family of insect endotoxin functioning in crop protection. As insects keep evolving into tolerance to the existing Crys, it is necessary to discover new Cry proteins to overcome potential threatens. Crys possess three functional domains at their N-termini, and the most active region throughout evolution was found at the domain-III. We swapped domain-IIIs from various Cry proteins and generated seven chimeric proteins. All recombinants were expressed in *Escherichia coli* and their toxicity was assessed by dietary exposure assays. Three of the seven Crys exhibited a high toxicity to Asian corn borer over the controls. One of them, Cry1Ab-Gc, a chimeric Cry1Ab being replaced with the domain-III of Cry1Gc, showed the highest toxicity to rice stem borer when it was over-expressed in *Oryza sativa*. Furthermore, it was also transformed into maize, backcrossed into commercial maize inbred lines and then produced hybrid to evaluate their commercial value. Transgenic maize performed significant resistance to the Asian corn borer without affecting the yield. We further showed that this new protein did not have adverse effects on the environment. Our results indicated that domain III swapped of Crys could be used as an efficient method for developing new engineered insecticidal protein.

## Introduction

Lepidopteran plant pests are a major factor in the yield loss of important crops^1–4^. The development of genetically modified crops expressing genes derived from *Bacillus thuringiensis* (BT) that encode insecticidal crystalline (Cry) proteins has proven to be effective in controlling these insects^5,6^. Many Cry-expressing varieties have been successfully commercialized to date, such as Bollgard II (expressing Cry1Ac and Cry2Ab) in cotton^7^ and Genuity-SmartStax (expressing Cry1A.105, Cry1F, Cry2Ab, Cry34Ab, and Cry35Ab) in maize^8^. The commercialization of insect-resistant crops leads to the increasing concern that insect populations are very likely to develop resistance to insecticidal proteins from these crops. Several methods have been suggested for the prevention of this problem. One is using high-dose Cry proteins in combination with a refuge^9^; another is mixing different toxins in one crop^10^. Exploring new insect-resistance proteins is another important approach to address emerging resistance^11–16^.

Cry toxins have been isolated from the natural environment, using traditional strategies of strain screening and molecular cloning. Based on sequence homology and insecticidal specificity, crystal proteins have been categorized into 68 main groups. In previous studies, the action mode of crystal proteins has been elucidated. Most Cry family proteins are considered to be composed of two distinct structural fragments, N-terminal and C-terminal. N-terminal is highly conserved and functions in toxicity, and C-terminal mainly acts to maintain the correct conformation of proteins^17^. Some evidence has supported that only overexpressing N-terminal fragments provide efficient insect-resistance in transgenic crops, such as MON810 (Monsanto)^8^. Different subfamilies of Cry proteins have been proved to have different spectra of activities on various insect targets, depending on their diverse amino acids in N-terminal fragments^18,19^. In N-terminal fragment of most Cry proteins, three key domains were identified. Domain I is composed of seven antiparallel α-helices and is involved in membrane pore formation. Domain II is composed of three beta-laminates and participates in the specific binding of intestinal receptors^20^. Domain III has a beta-sandwich structure, together with domain II, is responsible for structural stability and interaction with insect receptors^21^. Active toxins bind receptors at the brush border membrane of mid-gut insect cells, inducing pore formation and cell death.

Engineered domain-IIIs of Cry proteins have been suggested as promising tool strategies for the development and exploration of new insecticidal proteins^22^. The most famous case is Cry1A.105, which has been successfully used in commercial transgenic event Mon89034 (Monsanto). Cry1A.105 protein, which was constructed by domain I and domain II of Cry1Ab, domain III of Cry1F, and the C -terminal of Cry1Ac, improves the insecticidal effects against lepidopteran insects, especially fall armyworm^23^. Moreover, when domain III of Cry1Ab is substituted with domain III of Cry1C, the variant Cry1Ab toxin exhibits a six-fold enhancement of its activity against *Spodoptera exigua* compared with Cry1C^24^. Similarly, the exchange of domain III of Cry1Ia with that of Cry1Ba results in a toxin, exhibiting a seven-fold increase in the activity against the Coleopteran *Leptinotarsa decemlineata* compared with Cry1Ba^25^. Cry1Ab is toxic to Lepidopteran insects, and introduction of its domain III into Cry3Aa toxin provides effective in vitro activity against *Diabrotica virgifera* (Coleoptera: *Chrysomelidae*)^26,27^.

In this report, we examined 19 representative Cry proteins and analyzed their genetic relationships in a phylogenetic tree. Then we generated seven artificial Cry proteins by domain-swapping: we used Cry1Ab, the widely and commercially applied protein, as the domain I and II donors, and seven proteins from different subfamilies as domain III donors. These Cry proteins were expressed in *E. coli*, and we performed dietary exposure assays to assess their toxicity to insect pests. Our results showed that three of the artificial hybrid Cry proteins had high toxicity to Asian corn borer in both dietary exposure assay and transgenic rice. Furthermore, we selected the most toxic protein, Cry1Ab-Gc to analyze its ability to confer insect resistance, to assess its ecological risks, and to explore its potential commercial breeding value in maize.

## Results

### Design and synthesis of novel Cry proteins

**Figure 1:**
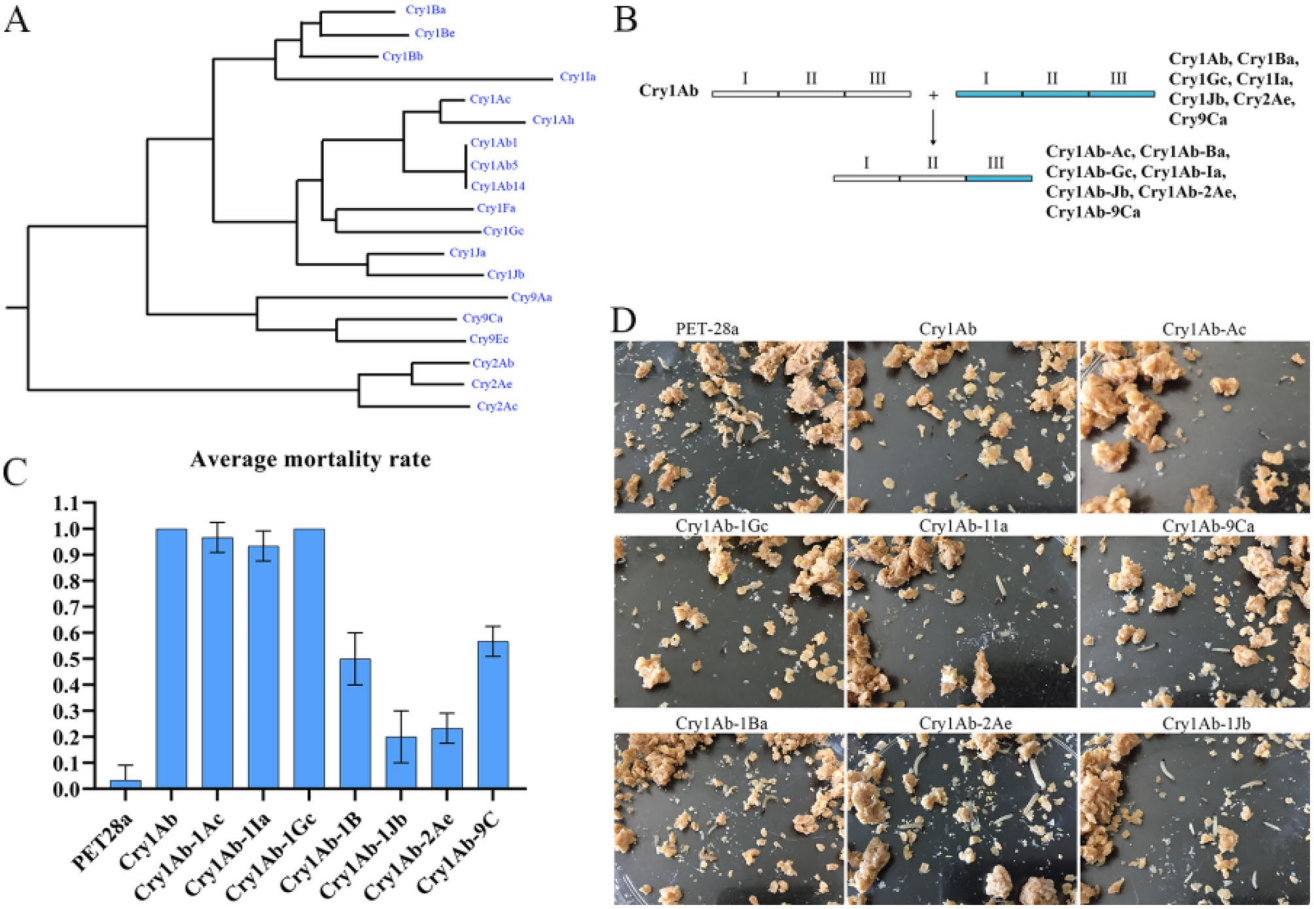
Molecular design and insect bioassay of new Cry proteins. (**A**) The phylogenetic relationship among Cry proteins. (**B**) Domain swapping is used to develop new Cry proteins. (**C**) Average mortality rates of Asian corn borer feeding on different Cry proteins. Vertical bars indicate mean ± SD (standard deviation) 5 days post-infestation. (**D**) The performance of Asian corn borer feeding on different Cry proteins.

We obtained 19 Cry protein sequences from National Center for Biotechnology Information (NCBI) database and analyzed their genetic relationship using DNAMAN software. Our phylogenetic analysis of Cry toxins indicated that the branch formed by Cry1 toxins was clearly and sufficiently separated from other toxins, classifying Cry1 toxins as a subdivision group in the phylogenetic tree (Fig. 1A). Moreover, Cry9 and Cry2 toxin groups were not located in Cry1 branch, but Cry9 toxins were more closely related to Cry1 toxins (Fig. 1A).

Seven representative Cry proteins, Cry1Ac, Cry1Ba, Cry1Gc, Cry1Ia, Cry1Jb, Cry1Ae, and Cry9Ca, were selected based on their phylogenetic relationships from 45 to 78% according to their amino acid sequence similarities. Seven new constructs were made by swapping domain III of the Cry1Ab protein for domain III of each selected Cry protein (Fig. 1B), and they were named based on domain III fusion. For example, Cry1Ab-1Gc has domains I and II from Cry1Ab and domain III from Cry1Gc. DNAMAN software was used to develop an exact multiple sequence alignment for different protein domain IIIs sequences. The identity of domain III of eight Cry proteins was 52.16% found by contrast, indicating that these domain-swapping engineered proteins were new Cry proteins (Supplementary Fig. 1).

### Testing Hybrid Proteins for Insecticidal Activity by Dietary exposure assay

To test the insecticidal activity of these hybrid proteins, we performed laboratory bioassays, using a diet incorporation method^28^. Results of the bioassays are illustrated in Fig. 1C and D. The column indicates relative levels of Asian corn borer activity. In the current study, the engineered hybrid Cry proteins displayed diverse toxicity to Asian corn borer larvae. Cry1Ab-1Jb and Cry1Ab-2Ae produced lower activities against Asian corn borer, resulting in lower mortality rates than others (Fig. 1D). Similar to Cry1Ab, Cry1Ab-1Gc, Cry1Ab-1Ac and Cry1Ab-1Ia showed higher toxicity against Asian corn borer than others (Fig. 1D). Insect mortality was the greatest on diets containing Cry1Ab-1Gc or Cry1Ab (Fig. 1C). These results indicated that Cry1Ab-1Gc had considerably similar toxicity to Asian corn borer by substituting Cry1Ab’s domain III with that of Cry1Gc.

### Expression and insect resistance analysis of Cry1Ab-1Gc, Cry1Ab-1Ac, and Cry1Ab-1Ia in transgenic rice

Cry1Ab-1Gc, Cry1Ab-1Ac, and Cry1Ab-1Ia were transformed to rice (cv. Jijing88) to determine their insecticidal activity in plants. To improve expression in plants, coding sequence (CDS) of engineered hybrid insecticidal protein was optimized codon usage for plant-preference, and *Ubi* promoter was used to effectively drive transgenic constitutive expression in monocot plants^29^ (Supplementary Fig. 2). 11 *Cry1Ab-Gc*, 15 *Cry1Ab*-*Ac*, 21 *Cry1Ab*-*Ia* independent over-expression (OE) events were obtained (Supplementary Fig. 3). The insect bioassay in the greenhouse showed that larvae of the rice stem borer were killed, and no bite holes were visible in the transgenic stems (Supplementary Fig. 4). The average mortality rate of rice stem borer feeding on transgenic rice was significantly higher than that of the control rice line (Jijing88) (Supplementary Table S1). All the transgenic rice showed significant enhancement rice stem borer resistance, for instance, *Cry1Ab*-*Gc* over-expression rice, which has the highest insecticidal activity to rice stem borer.

**Figure 2:**
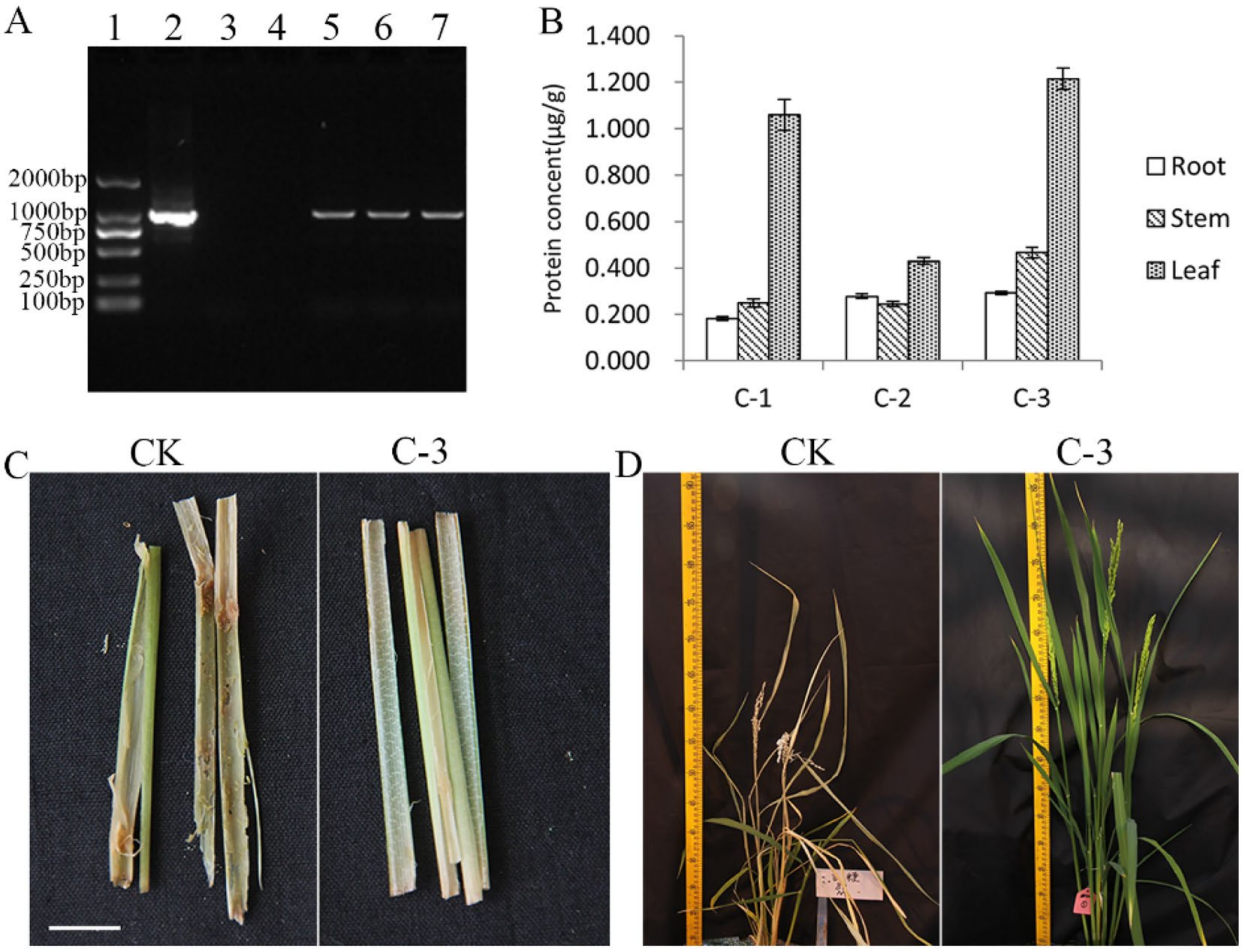
Expression and insect resistance analysis of Cry1Ab-Gc protein in rice. (**A**) PCR analysis of rice genomic DNA. 1: DL 2000 Marker; 2: Plasmid positive control; 3: Black control; 4: Negative Control; 5: Transgenic event C-1; 6: Transgenic event C-2; 7: Transgenic event C-3. (**B**) ELISA of Cry1Ab-Gc protein in three transgenic events. (**C**) Insect resistance performance of rice stems in the laboratory. Scale bar, 1 cm. (**D**) Insect resistance performance in the glasshouse.

Figure 2A shows the PCR result of a representative *cry1Ab-1Gc* gene amplification product (Fig. 2A and Supplementary Fig. 8). ELISA data showed that Cry1Ab-1Gc protein was present in three independent transgenic events, and C-3 event had higher Cry1Ab-1Gc protein content (Fig. 2B). All transgenic events had no obvious damage symptoms, the control rice showed dead sheaths, withered white particles, and a greater number of tillers with damaged borders. Figure 2C,D and Table 1 displayed the result of insect resistance assessment of representative C-3 line. The damaged tiller rate (number of tillers damaged/total tillers) in the transgenic rice was 3.95%, which was substantially lower than that observed in the control rice (46.15%). These results show that transgenic rice expressing the Cry1Ab-1Gc toxin exhibited higher resistance against lepidopteran insects.

**Table 1:** Insect resistance assay of the transgenic rice in the greenhouse.

Rice lines	No. of plants	Total tillers	No. of tillers damaged	Damaged tiller rate (%)
CK	45	832	384	46.15 ± 4.31**
C-3	45	810	32	3.95 ± 0.44

Means (± SE) within a column followed by different letters are significantly different (T-test, **P < 0.01).

### Insect resistance assay of Cry1Ab-Gc in transgenic maize

**Figure 3:**
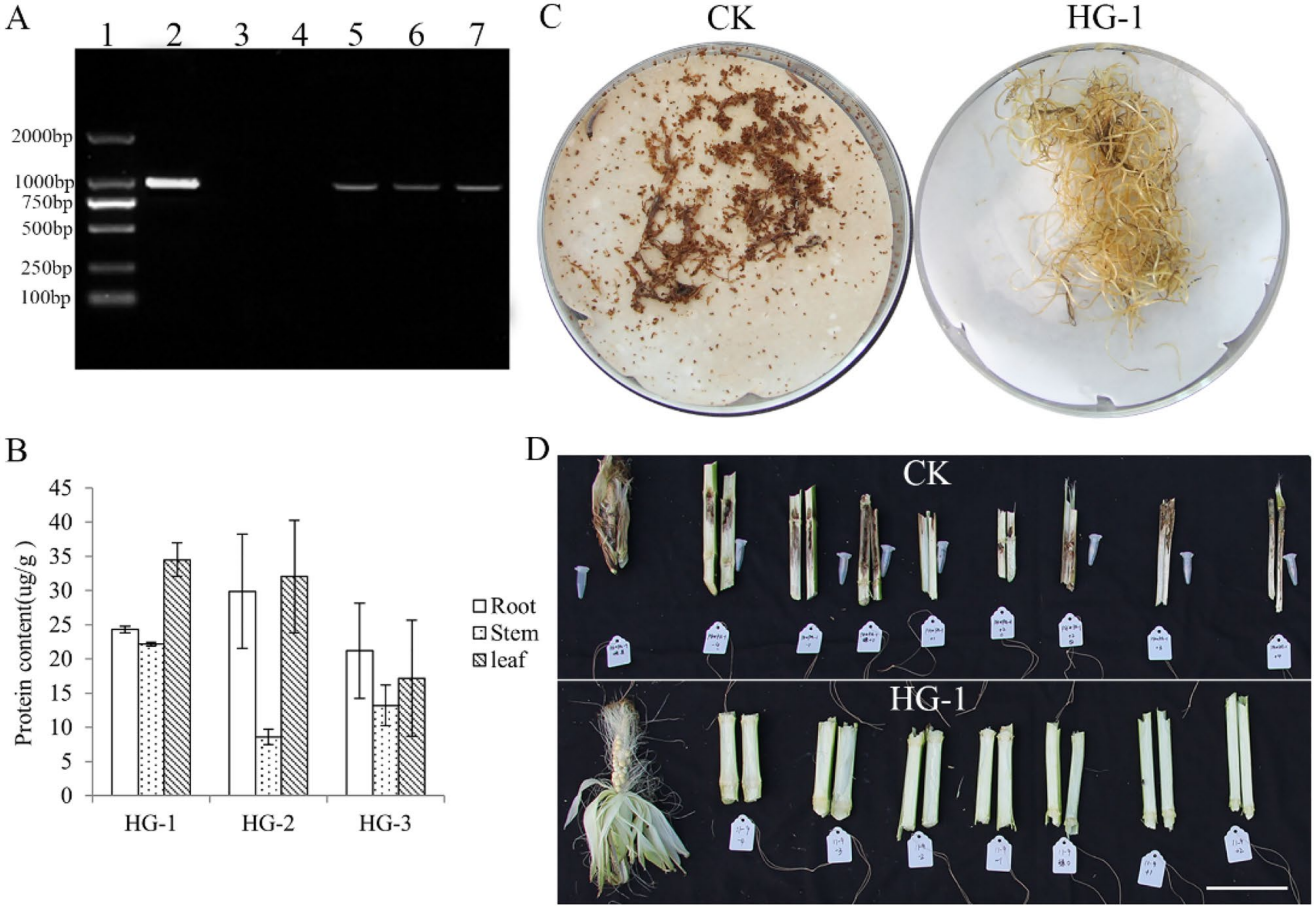
Expression and insect resistance analysis of Cry1Ab-Gc protein in maize. **(A**) PCR analysis of maize genomic DNA. 1: DL 2000 Marker; 2: Plasmid positive control; 3: Black control; 4: Negative Control; 5: Transgenic event HG-1; 6: Transgenic event HG-2; 7: Transgenic event HG-3. (**B**) ELISA of Cry1Ab-Gc protein in three transgenic lines. (**C**) Insect resistance performance of maize filament in laboratory. (**D**) Insect resistance performance of maize in field. Scale bar, 10 cm.

To further analyze commercialization of Cry1Ab-1Gc, the plant expression vector pTF101.1-Cry1Ab-1Gc was transferred into maize (*cv. HiII*) by *Agrobacterium*-mediated transformation. We selected three transgenic events (HG-1, HG-2, and HG-3) for assessment. The results of molecular experiments showed that *cry1Ab-Gc* gene was stably integrated into maize (Fig. 3A and Supplementary Fig. 9). ELISA data showed that HG-1 event had higher Cry1Ab-Gc protein content than others on the leaf, root, and stem (Fig. 3B).

The number of borers, the length of borer tunnels, and the number of surviving larvae were recorded at the silking stage in a function identification pool. The result showed that three transgenic events had good insect resistance. Event HG-1 was the best one, the number of borers in transgenic maize HG-1 was fewer, the length of tunnels was shorter (Fig. 3D and Table 2). There were almost no live insects, but there were more live borers in non-transgenic maize, and a significant difference between them was found (Supplementary Table S2). As illustrated in Fig. 3C, event HG-1 had good resistance, as little filaments were fed, and the corn borer was dead after receiving transgenic maize filament in a laboratory bioassay.

**Table 2:** Insect resistance of transgenic inbred lines against Asian corn borer.

Maize lines	No. of plants	No. of channels	Length of tunnel (cm)	No. of live larvae
CK	12	2.92 ± 0.62	4.75 ± 1.23	0.75 ± 0.25
HG-1	14	0.36 ± 0.17**	1.36 ± 1.00**	0.00 ± 0.00**

Means (± SE) within a column followed by different letters are significantly different (*t*-test, **P < 0.01).

**Figure 4:**
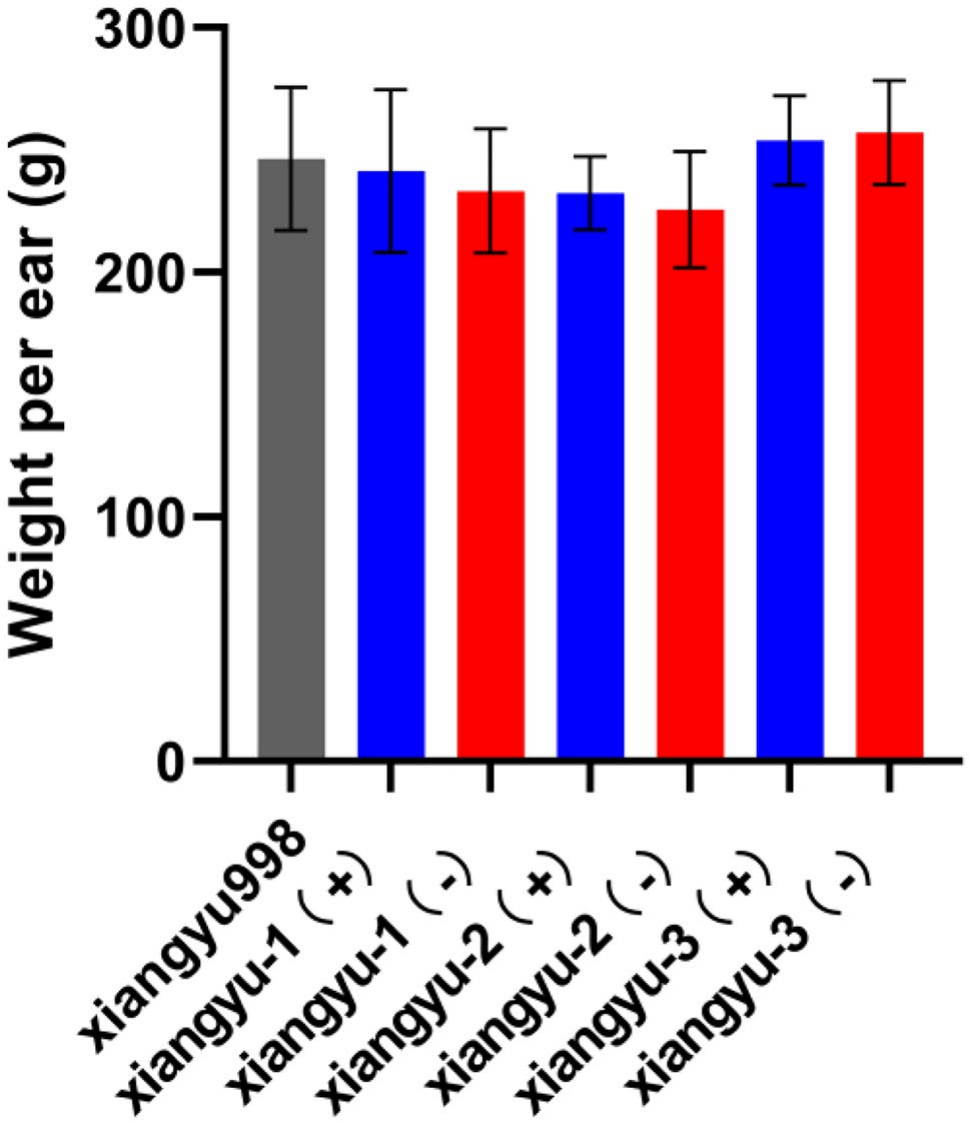
Weight per ear of different hybrids compared with commercial varieties. xiangyu-1( +), xiangyu-2( +), xiangyu-3( +): Transgenic hybrids; xiangyu-1(-), xiangyu-2(-), xiangyu-3(-):Negative separation controls.

To further evaluate the application value of Cry1Ab-1Gc, event HG-1 was backcrossed to local commercial inbred line Y822 and Ji853. It took us four years to obtain the commercial transgenic maize inbred lines of BC_5_F_5_ generation (the detailed breeding information is presented in Supplementary Fig. 10), and their genetic background was almost the same as that of recurrent varieties. In the field, the Asian corn borer caused no visible damage to transgenic commercial inbred lines. However, the control plants infested by Asian corn borer showed significantly greater numbers of holes and longer tunnels (Table 3). Our results reveal that *cry1Ab-Gc* transgenic maize has higher resistance to Asian corn borer than non-transgenic maize plants. Furthermore, three independent backcross inbred line of Y822_HG-1_ were selected as the female parents, and crossed with the male parent X923-1 to product the hybrid seed Xiangyu998_HG-1_. The field test results showed that there was no significant difference in yield traits under commercial hybrid background (Fig. 4), implying that cry1Ab-Gc has a good commercial potential in the future.

**Table 3:** Insect resistance of transgenic inbred lines (Ji853 ^Cry1Ab-Gc^ and Y822 ^Cry1Ab-Gc^) against Asian corn borer.

Maize lines	No. of plants	No. of channels	Length of tunnel (cm)	No. of live larvae
Ji853	13	1.15 ± 0.27	2.77 ± 0.90	0.23 ± 0.17
Ji853 ^Cry1Ab-Gc^	24	0.25 ± 0.09**	0.35 ± 0.25**	0.08 ± 0.06*
Y822	13	2.46 ± 0.55	5.00 ± 1.40	0.54 ± 0.27
Y822 ^Cry1Ab-Gc^	12	0.58 ± 0.19**	1.00 ± 0.44**	0.08 ± 0.08**

Means (± SE) within a column followed by different letters are significantly different (*t*-test, **P < 0.01,* *P* < 0.05).

Considering that Cry1Ab-Gc is a new BT protein, we then performed an environmental impact to evaluate and analyze in the field. In order to further clarify whether genetically modified corn is harmful to the environment, population dynamics and biodiversity of the non-target arthropod (a representative environmental indicator) were investigated and analyzed by direct observation and pitfall traps. Temporal variation of biodiversity in one year supported that there were no significant differences between Cry1Ab-Gc maize and the non-BT maize field in the total number of individuals of the dominant arthropod species, the Shannon index (H), Pielou index (J), and Simpson index (D) (Supplementary Fig. 5). Field tests also show that Cry1Ab-Gc has no significant negative impact on the population dynamics of arthropod community (Supplementary Figs. 6–7), suggesting that this new engineered hybrid insecticidal protein Cry1Ab-Gc hold no significant impact on ecology.

## Discussion

Insects resistant to Cry proteins have developed in the field, which has successfully revolutionized modern agriculture^30^ With the large-scale planting of transgenic crops, insects keep evolving into tolerance to the existing Crys. To control Bt-resistant insects, the strategy of the second generation insect protected, which expresses multiple Bt proteins, Cry1Ac, Cry1A.105 and Cry2Ab2, or Cry1A and Cry1F, were commercialized in crop biotechnology fields^25–27^. However, to overcome potential threats from the development of resistant insect populations and further protect the long-term utility of BT technology of pest control, new Crys are in continual demand as insects keep evolving into tolerance to the existing Crys. Cry proteins vary in their toxicity to different insects, and determine such differences can inform efforts to produce transgenic insect-resistant crops. Insects can distinguish and preferentially feed on diets containing different Cry proteins^31,32^. Cry proteins have a diversity of insecticidal activities to different lepidopteran insects, including *Earias insulana* (Lepidoptera: Noctuidae), *Lobesia botrana* (Lepidoptera: Tortricidae)^33^, *Chilo suppressalis* (Lepidoptera: Pyralididae)^19^, *Cnaphalocrocis medinalis* (Lepidoptera: Pyralidae)^34^. These results support that the continuous evolution of Cry proteins is the key to produce different resistance and different insect resistance profiles. Crys possess three functional domains at their N-termini^35,36^, Our bioinformatics studies showed that the most active region throughout the evolution are found at the domain-III (data not shown). We swapped domain-IIIs from various Cry proteins and generated seven chimeric proteins, aiming to overcome the insect tolerance. In previous study, it has shown that domain recombination is an important strategy to create new Bt proteins^37^. For example, Bayer CropScience has constructed two new Bt fusion proteins, Cry1A.2 and Cry1B.2. Cry1A.2 contains domain I of Cry1Ah, domain II of Cry1Ac, domain III of Cry1Ca and protoxin domain of Cry1Ac; Cry1B 0.2 contains domain I and domain II of Cry1Be2, domain III of Cry1Ka, and the protoxin domain of Cry1Ab3. Studies have confirmed that specific domain recombination can produce higher insecticidal activity against some key lepidopteran pests and reduce the probability of resistant insect populations^38^.

Our objective in this study is to test the ability of domain III exchange alone to produce new insect-resistant proteins and to evaluate whether combinations of different subfamilies of proteins have different effects. We selected seven distantly related Cry proteins and constructed seven novel domain mutants by swapping domain III of Cry1Ab with seven related proteins. We then used dietary exposure assays to characterize the toxicity of new mutant Cry proteins to Asian corn borer resulting in the following insect resistance ranking: Cry1Ab-1Gc ≧ Cry1Ab > Cry1Ab-1Ia > Cry1Ab-1Ac > Cry1Ab-9Ca > Cry1Ab-1Ba > Cry1Ab-2Ae > Cry1Ab-1Jb > pET28a. These results indicated that not all domain IIIs exchanges can improve the insecticidal effect of the antibacterial protein. The exchange and recombination of 1Ab with 1Gc, 1Ia, 1Ac have better insect resistance than other combinations. Similar studies have been reported before, Cry1A/Cry1I-like fusion protein expressed in transgenic rice is effective at controlling lepidopteran pests^39^. Interestingly, in our study, we also found engineered hybrid Cry1Ab and Cry1Ia provided new insecticidal proteins for pest management in rice. This suggested that domain III swaps of Cry1A with other Cry1I gene subfamily member may also discover more novel insect resistance genes. Among the multiple recombinant proteins, the most active ones are the recombinant proteins of Cry1Gc and Cry1Ab. Cry1Gc, also named CryET66, showed insecticidal activities against the European corn borer, but its toxicity was lower than that of Cry1Ab^40^. Interestingly, according to our results, the exchange of domain III of Cry1Gc with Cry1Ab improved Cry1Gc’s resistance to the Asian corn borer, demonstrating similar insecticidal activities with Cry1Ab. Our findings suggest that the main reason for the low virulence of Cry1Gc is likely to come from its domain I and domain II, while domain III of Cry1Gc probably only plays an important role in recognizing receptors. Furthermore, we also speculate that the combination of domain I and II of Cry1Ab and domain III of Cry1Gc may increase the stability of expression proteins, thereby enhancing insect resistance. In addition, the recognition of Cry toxins by cell surface receptors has been proven to occur in different domain III regions^41–44^. Cry1Ab and Cry1Gc shared 53.96% identity in domain III. It has higher insecticidal activity than Cry1Ab protein to *Plutella xylostella* (Lepidoptera: Plutellidae)^40^. Therefore, Cry1Ab-1Gc engineered hybrid insecticidal proteins may exhibit a broader insecticidal spectrum. In the future, we aim to explore whether protein confers resistance to other insects, its cross-resistance, and its specific insect binding site. The relationship of these residues with insect resistance will be evaluation require further examination of 3D protein structure and anti-insect mechanism *via in silico* analyses^45^.

In addition to the discovery of new recombinant proteins, this study further evaluated the breeding value of the obtained new genes in crops. Cry1Ab-1Gc was selected for more intensive studies in crops. Rice is one of the most important crops in the world and a model monocot system. Insect resistance bioassays in a greenhouse demonstrated that the transgenic rice created in the current study was highly resistant to rice stem borer. In addition, Asian corn borer seriously affected the quality and yield of spring maize in Northeast China^46^. Jidan209 and Xiangyu998 are commercial maize hybrids with high yields and disease resistance, but susceptible to damage from insects. In our study, Cry1Ab-Gc over-expression event was backcrossed and transferred to Ji853 (female parent of Jidan209) and Y822 (female parent of xiangyu998). Field bioassays indicated that Cry1Ab-Gc can significantly confer sufficient resistance to Asian corn borer in maize commercial inbred line. Using Y822 as the female parent and X923-1 as the male parent, three transgenic hybrid combinations were prepared, and the yield of the transgenic hybrid combination and the commercial variety Xiangyu 998 was tested. The results showed that the transgenic *cry1Ab-Gc* gene hybrid combination conferred insect resistance to maize, but the weight per ear is not significantly different from Xiangyu 998. Our study provides a new report of insect resistance produced by the expression of a novel engineered hybrid insecticidal protein in both rice and different maize inbred lines in China.

Furthermore, although BT crops have been commercially planted in a large number of countries, their safety for environment is still controversial^47^. However, this safety of new BT protein is the key to commercial application. In this study, the new engineered hybrid insecticidal protein Cry1Ab-Gc was performed the ecological risk assessment in transgenic maize with one-year field investigation by diverse methods. We selected non-target arthropods as an important environmental indicator based on their sufficient abundance, low mobility, and clear path of exposure^48^. Our results showed the Cry1Ab-Gc over-expression maize had no significantly negative impacts on arthropod diversity in the field. This result was consistent with previous studies, which proved that BT maize include Cry1Ab^49^, Cry1Ac^50,51^ and Cry3Bb^52^ did not affect the population dynamics and biodiversity of the non-target insect. Notably, in this study, we only conducted one-year research, so continuous and systematic research would be helpful to confirm the environmental safety of Cry1Ab-Gc protein release.

## Conclusion

In summary, we constructed seven new Cry proteins by a domain-swapping strategy, and confirmed that exchanging the domain III of Cry proteins alone can produce new insect-resistant toxin proteins. In this study, three high insect-resistance new proteins were discovered, among which Cry1Ab-Gc shown highest resistant to two lepidopteran insects, rice stem borer and Asian corn borer, when it was overexpressed in rice and maize. The result of ecological risk assessments suggests the release of Cry1Ab-Gc protein did not have adverse effects on the environment, supporting that Cry1Ab-Gc can provides a new tool for Lepidopteran insects resistant management in crop protection.

## Methods

### Insects

Neonates of Asian corn borer (*Ostrinia furnacalis*) and Rice stem borer (*Chilo suppressalis*) used in this study were obtained from the General Pest Company (Beijing, China) and were kept in a climate-controlled chamber at 26 ± h °C, 70 ± 7mber at 2 s study were obtained from the General6:8 (L: D) h at Agro-biotechnology Research Institute, Jilin Academy of Agricultural Sciences, China.

### Design and synthesis of novel Cry proteins

The different Cry protein sequences,

Cry1Ab1 (https://www.ncbi.nlm.nih.gov/protein/AEV45790.1),

Cry1Ab5 (https://www.ncbi.nlm.nih.gov/protein/CAA28405.1),

Cry1Ab14 (https://www.ncbi.nlm.nih.gov/protein/AAG16877),

Cry1Ac (https://www.ncbi.nlm.nih.gov/protein/WP_000369821.1),

Cry1Ah (https://www.ncbi.nlm.nih.gov/protein/AAQ14326.1),

Cry1Ba (https://www.ncbi.nlm.nih.gov/protein/WP_000203376.1/),

Cry1Be (https://www.ncbi.nlm.nih.gov/protein/O85805.1),

Cry1Bb (https://www.ncbi.nlm.nih.gov/protein/WP_000203375.1),

Cry1Ia (https://www.ncbi.nlm.nih.gov/protein/CAA44633.1),

Cry1Fa (https://www.ncbi.nlm.nih.gov/protein/Q03746.1),

Cry1Ja (https://www.ncbi.nlm.nih.gov/protein/WP_098369107.1),

Cry1Jb (https://www.ncbi.nlm.nih.gov/protein/Q45716.1),

Cry9Aa (https://www.ncbi.nlm.nih.gov/protein/WP_087997148.1),

Cry9Ca (https://www.ncbi.nlm.nih.gov/protein/Q45733.1),

Cry9Ec (https://www.ncbi.nlm.nih.gov/protein/AAS68357.1),

Cry2Ab (https://www.ncbi.nlm.nih.gov/protein/WP_001089638.1),

Cry2Ae (https://www.ncbi.nlm.nih.gov/protein/ABW49930.1),

Cry2Ac (https://www.ncbi.nlm.nih.gov/protein/Q45743.1),

Cry1Gc^40^ were obtained from the National Center for Biotechnology Information (NCBI) database, and the genetic relationships were analyzed using DNAMAN software^44^ (Version 6). Construct domain mutants were created by swapping domain III of seven distantly related proteins for domain III of Cry1Ab protein. The sequence of Domain I and II from Cry1Ab, Domain III from Cry1Ab and other 7 Cry proteins are shown in the supplementary file (Supplementary sequence 1–9). Optimized gene nucleotide sequences are also displayed in the supplementary file (Supplementary sequence 10–16).

### Dietary exposure screen

The coding sequences of all the designed new proteins were optimized by codon preference of *Escherichia coli* by Sheng Gong Company (Shanghai, China). The *E. coli* expression vector pET28a, which was digested with *Hind*III and *EcoR*I, was used as the vector backbone, and ligatedwith the artificially synthesized gene sequence by T4 DNA ligase (Thermo Fisher Scientific, USA), and then transformed into *E. coli* DH5α^53^. All recombinant proteins were expressed in *Escherichia coli* BL21 (DE3)plysS. The detection primers for each construct are shown in the supplementary file (Supplementary Table 3). *E. coli* clones that express seven Cry proteins, Cry1Ab-1Gc, Cry1Ab-1Ia, Cry1Ab-1Ac, Cry1Ab-9Ca, Cry1Ab-1Ba, Cry1Ab-2Ae, and Cry1Ab-1Jb were grown overnight. The expression vector pET28a without Cry protein was used as the negative control. 5 ml overnight culture was sonicated and the amount of protein to be tested. Stock solutions of the seven Cry proteins were diluted with distilled water and incorporated into an artificial diet for Asian corn borer to the same concentration, 100 μg/ml. Since the Asian corn borer diet must be heated during preparation, the Cry protein solutions were mixed into the diet when its temperature had decreased to < 60 °C to avoid degradation. Once the food was solid, it was cut into slices and individually placed in 10 cm diameter Petri dishes. Ten neonates of Asian corn borer were transferred into each Petri dish, which was then sealed with breathable film to prevent the larvae from escaping. The feeding assay was conducted in a growth chamber at 28 °C, 80% relative humidity, and a 12 h photoperiod. Three replicates were used for each protein. Mortality was recorded after 5 days.

### Construction of over-expression vector and genetic transformation

*Cry1Ab-1Gc, cry1Ab-1Ac, cry1Ab-1Ia*, the synthetic *cry* genes were designed as outlined through the codon usage of rice^50^, These coding sequences are shown in Supplementary sequence 17–19. The pTF101.1 vector and the plasmid containing *cry* genes were digested with *Sma*I and *Sac*I, and two fragments were connected to the vector by T4 DNA ligase (Thermo Fisher Scientific, USA). The Agrobacterium EH105 containing the above vectors were transformed into rice (*cv. Jijing 88*) by *Agrobacterium*-mediated genetic transformation, respectively^54^ After successful transformation, genomic DNAs were extracted by CTAB method^55^. PCR method was used for screening and verification. The detection primers for each gene are in the supplementary file (Supplementary Table 3). The reaction conditions were optimized and mixtures (20 μl total volume) were composed of 2 × 10 μl EasyTaq PCR SuperMix dNTPs, 0.2 μl primer (10 mmol/l), 1 μl DNA (150 ng), and 8.6 μl ddH_2_O. A Biometra PCR Thermal Cycler was used for amplification, programmed for 40 cycles as follows: 95 °C/5 min for primary denaturation (1 cycle); 95 °C/30 s for denaturation, 58 °C/30 s for annealing with *cry1Ab-Gc* primers, 72 °C/1 min for extension (30 cycles); 72 °C/10 min (1 cycle); 4 °C (overnight storage). Agarose (1.2%) gel electrophoresis was used for resolving PCR products. The Gene Ruler 2000-bp DNA ladder (Takara, Japan) was also included in gel electrophoresis, which was performed at 80 V in a submarine electrophoresis unit. Bands were detected on a UV-transilluminator and photographed. To test the toxicity of the new protein against insects in monocot crops, *cry1Ab-Gc*, was also transformed into maize (*cv. HiII*) according to the *Agrobacterium* transformation method reported in the literature^56^. The same PCR detection method was used to confirm the presence of the transgene is the same as rice detection method. The detection primers for each construct were in the supplementary file (Supplementary Table 3).

### Enzyme-linked immunosorbent assay (ELISA)

The expression of Cry1Ab-Gc protein in transgenic plants was quantitatively analyzed by Enzyme-linked immunosorbent assay (ELISA) using the Cry1Ab/Cry1Ac plate kit (Agida, USA) according to its instructions. Leaves of transgenic rice at the tillering stage were ground into powder using liquid nitrogen, and 0.02 g powder was weighed into 1.5 ml centrifuge tubes with 500 μl extraction buffer. Samples were diluted 400-fold after mixing, and 50 μl of each sample was used for detection. The non-transgenic rice (cv. Jijing 88) was used as a negative control. The standard curve was constructed using a concentration gradient (0.02, 0.016, 0.012, 0.008, 0.004, and 0 ng) for the quantitative analysis of samples. We selected one transgenic line (C-3) with high Cry1Ab-Gc protein levels for insect bioassay. The same method was used to confirm the presence of the transgene in maize.

### Evaluation of insect resistance of transgenic rice

The insect bioassay was conducted using the test tube method in the laboratory. Rice stems of three transgenic rice lines with *cry1Ab-Gc*, *cry1Ab-Ac* and *cry1Ab-Ia* at the tillering stage were cut into 6-cm sections. Five stem sections were each placed into separate test tubes, filled with 1% agar to a depth of 2 cm. Ten rice stem borer larvae were released into each test tube with 5 repetitions. Test tubes were sealed with cotton balls to prevent the larvae from escaping. The feeding assay was conducted in a growth chamber at 28 °C, 80% relative humidity, and 12-h photoperiod. Damage to stem tissues and larval mortality were observed and analyzed by *t-*test after infestation for five days. Non-transgenic plants were used as a negative control. All data were analyzed by *t*-test (*P < 0.05; **P < 0.01).

Transgenic rice event C-3 with *cry1Ab-Gc* gene was planted in a greenhouse with normal field treatment and surrounded with wild-type Jijing88 as a negative control. No pesticide was applied during the growth period. Twenty neonates of rice stem borer were placed into the hearts of tillers of every rice plant at the tillering stage. Insect occupancy was observed every 12 days. Rice tiller performance and the number of tillers damaged were compared with the controlled rice by *t*-test (*P < 0.05; **P < 0.01).

### Evaluation of insect resistance of transgenic maize

Cry1Ab-Gc was transformed into maize (*cv. HiII*) using *Agrobacterium*-mediated genetic transformation with pTF101.1-cry1Ab-Gc vector. Twenty neonates of Asian corn borer were placed into the heart leaf of each transgenic plant and the control plant at the silking stage in a function identification pool. The insect occupancy was observed after 14 days. Agronomic performance, length of the tunnels produced by larvae, and the number of survival larvae were observed and analyzed by *t*-test (*P < 0.05; **P < 0.01).

Event HG-1 was backcrossed to local commercial inbred lines Y822 and Ji853. F_1_ hybrid materials were obtained by event HG-1 as male parent and commercial inbred lines Y822 and Ji853 as female parent. Then, the F_1_ generation material obtained was used as the male parent and further backcrossed with the commercial inbred lines Y822 and Ji853 of the female parent material. After backcrossing for 5 generations, it was self-crossed for 5 generations, and finally the improved commercial version of insect resistant inbred line containing insect resistant gene was obtained for phenotypic identification. To the further, Cry1Ab-Gc expressing maize inbred lines cv. Ji853 and Y822 were assessed by the same method above. Agronomic performance and length of the tunnels were compared with the control by *t*-test (*P < 0.05; **P < 0.01).

### Hybrid yield test and statistical analyses

Xiangyu 998 is a commercial hybrid that uses Y822 as the female parent and X923-1 as the male parent. We crossed the Y822 transgenic inbred line with X923-1 to obtain Xiangyu998 transgenic hybrid combinations. We select three lines of transgenic hybrids xiangyu-1(+), xiangyu-2(+), xiangyu-3(+), and the corresponding negative materials, xiangyu-1(-), xiangyu-2(-), xiangyu-3(-). Each material plant 10 rows, each row plant 20 plants under natural rainfall conditions. All material plants were open pollination, and collected at maturity period. Measure the weight of each row corn ears, record the number of ears in each row, calculate the weight of each ear, and perform statistical analysis by *t*-test.

### Biodiversity investigation and statistical analyses

Cry1Ab-Gc maize and the non-BT maize (CK) were grown in the GM fields of Jilin Academy of Agricultural Sciences located in Jilin province (2020; China; E125°, N44°). Cry1Ab-Gc maize was divided into two groups, spray herbicide glufosinate (F-P) and none glufosinate application (F-BP). The number of arthropods was investigated and analyzed by direct observation and pitfall traps method^57^. Six species of arthropods (*Harmonia axyridis*, ladybird larvae, aphids, *Monolepta hieroglyphica*, spiders, and *Propylaea japonica*) were counted and analyzed by the method of direct observation; Four species of arthropods (*Teleogryllus infernalis*, Earwig furficulidae, Opiliones, and *Carabidae sp.4*) were counted and analyazed by the method of pitfall traps. Three indices were used to analyze the dynamics of the arthropod community: Shannon index^58^, Pielou index ^59^, and Simpson index^60^.

### General declaration

The plant collection and use was in accordance with all the relevant guidelines.

## Supplementary Information

Supplementary Information.
