# Supplementary material for: Engineered chimeric insecticidal crystalline protein improves resistance to lepidopteran insects in rice (Oryza sativa L.) and maize (Zea mays L.)
Source: Sci Rep. 2022 Jul 22;12:12529. doi: 10.1038/s41598-022-16426-6 (PMC9307649; doi:10.1038/s41598-022-16426-6)
Supplement: Supplementary file 1 — Supplementary Information. [file 41598_2022_16426_MOESM1_ESM.docx]

Engineered chimeric insecticidal crystalline protein improves resistance to lepidopteran insects in rice (*Oryza sativa L.*) and maize (*Zea mays L.*)

Yang Liu^1†^, Siping Han^1†^, Shuo Yang^2^, Ziqi Chen^1^, Yuejia Yin^1^, Jinghui Xi^3^, Qing Liu^1^, Weiyu Yan^1^, Xinyuan Song^1^, Fangfang Zhao^4^, Jia Guo^1^, Xiangguo Liu^1*^, Dongyun Hao^1*^

^1^Institute of Agricultural Biotechnology, Jilin Academy of Agricultural Sciences, Changchun, China

^2^College of Life Science, Jilin University, ChangChun, China

^3^College of Plant Science, Jilin University, ChangChun, China

^4^College of Life Science and Technology, Harbin Normal University, Harbin, China

Yang Liu and Siping Han contributed equally to this manuscript

*** Correspondence:** Xiangguo Liu: lxgyyj@cjaas.com; Dongyun Hao: [dyhao@cjaas.com](mailto:dyhao@cjaas.com)

**Supplementary Figure 1.** Multiple amino sequence alignment of domain III from 7 Cry proteins and Cry1Ab protein


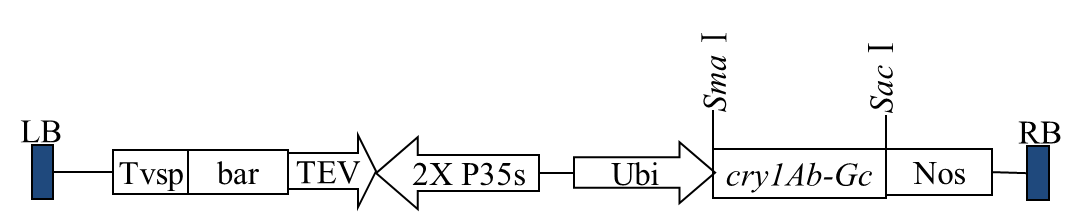


**Supplementary Figure 2.** A schematic diagram of the T-DNA region of the plant expression vector pTF101.1-cry1Ab-1Gc. Tvsp: terminator; *bar*: resistance gene encoding sequence; TEV: enhancer; *2×P35s: double cauliflower mosaic virus* (CaMV) 35S promoter; Ubi: maize *ubiquitin* promoter; *Cry1Ab-1Gc*: *cry1Ab-1Gc* gene encoding sequence; Nos: nopaline synthase terminator.

**
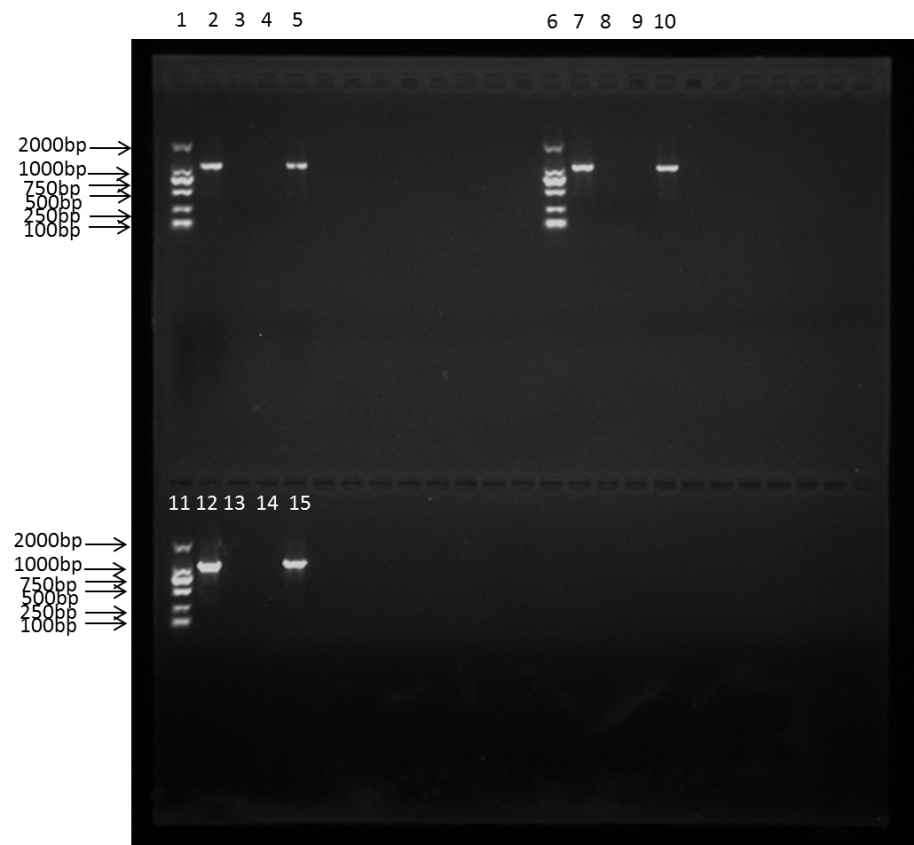
**

**Supplementary Figure 3** PCR detection of three *cry* genes in transgenic rice. 1, 6 and 11: DL 2000 Marker; 2: Plasmid positive control of *cry1Ab-Ac* gene; 3: Black control; 4: Negative Control; 5: *cry1Ab-Ac* OE rice. 7: Plasmid positive control of *cry1Ab-Ia* gene. 8: Black control; 9: Negative Control; 10: *cry1Ab-Ia* OE rice. 12: Plasmid positive control of *cry1Ab-Gc* gene. 13: Black control; 14: Negative Control; 15: *cry1Ab-Gc* OE rice.


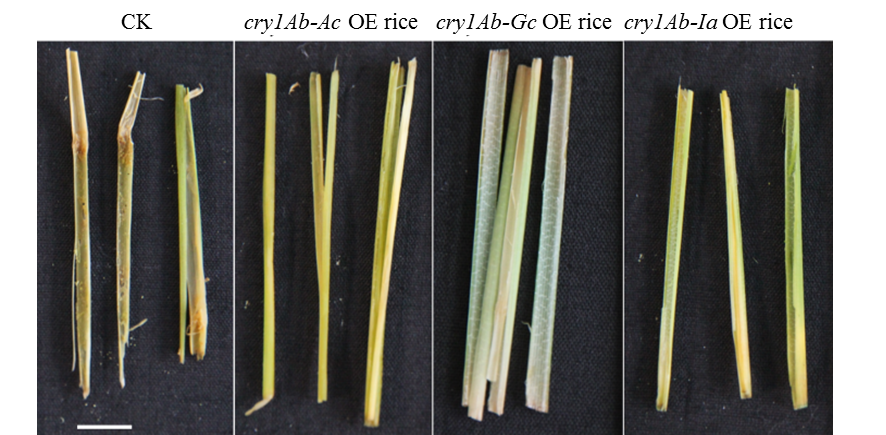


**Supplementary Figure 4.** Insect resistance performance of rice stems in the laboratory. CK: Jijing 88; *cry1Ab-Ac* OE rice: transgenic *cry1Ab-Ac* rice; *cry1Ab-Gc* OE rice: transgenic *cry1Ab-Gc* rice; *cry1Ab-Ia* OE rice: transgenic *cry1Ab-Ia* rice. Scale bar, 1 cm


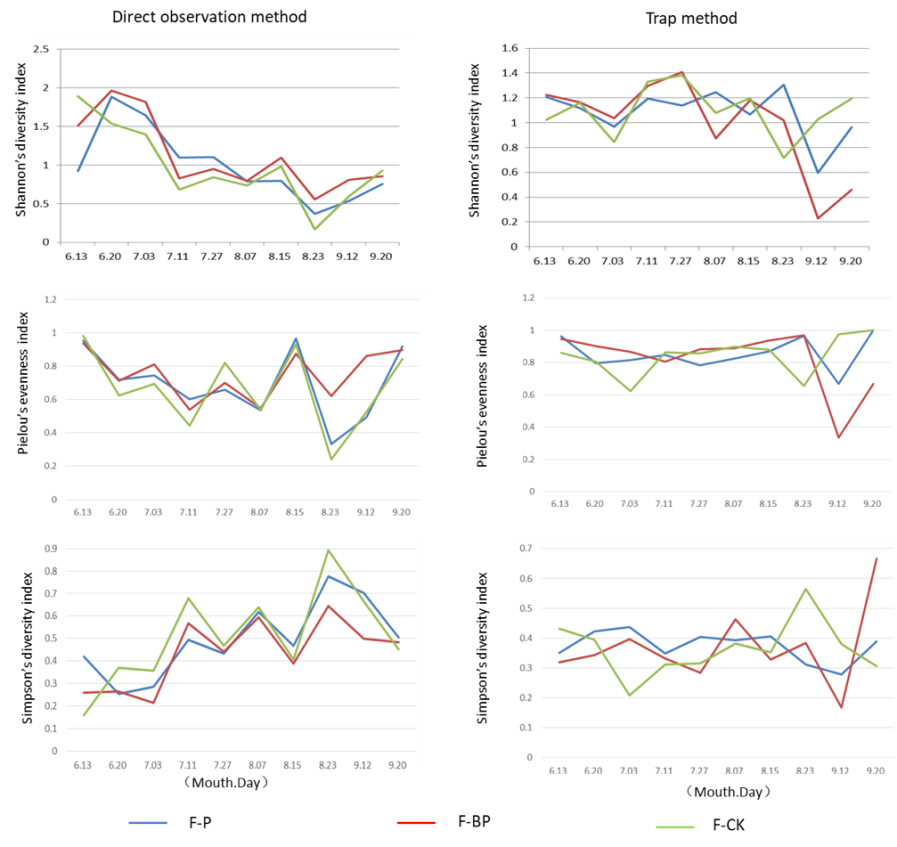


**Supplementary Figure 5.** Effects on arthropod community structure by direct observation method and pitfall trap method. The abscissa represents survey time. Three indices were used to analyze the dynamics of the arthropod community: Shannon’s diversity index, Pielou's diversity index, and Simpson's diversity index.

**
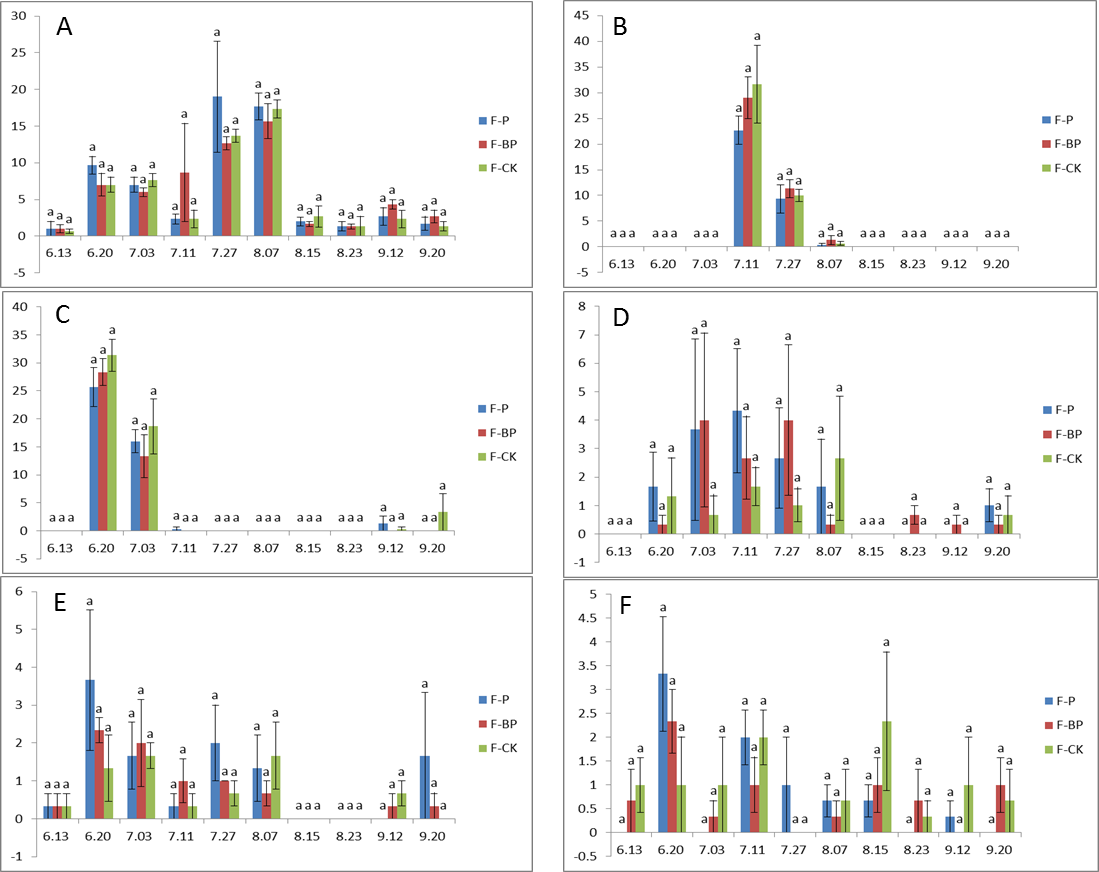
**

**Supplementary Figure 6.** Effects on arthropod community structure (direct observation method). (**A**) *Harmonia axyridis*; (**B**) ladybird larvae; (**C**) aphids; (**D**) *Monolepta hieroglyphica*; (E) spiders; (F) *Propylaea japonica*. F-CK: non-Bt maize control; F-P: Cry1Ab/Gc maize spraying herbicide glufosinate; F-BP: Cry1Ab/Gc maize with no glufosinate application. The ordinate represents the average number of individuals in each community ± SD, and the abscissa represents survey time. Statistically significant differences according to one-way ANOVA (a: P>0.05).

**
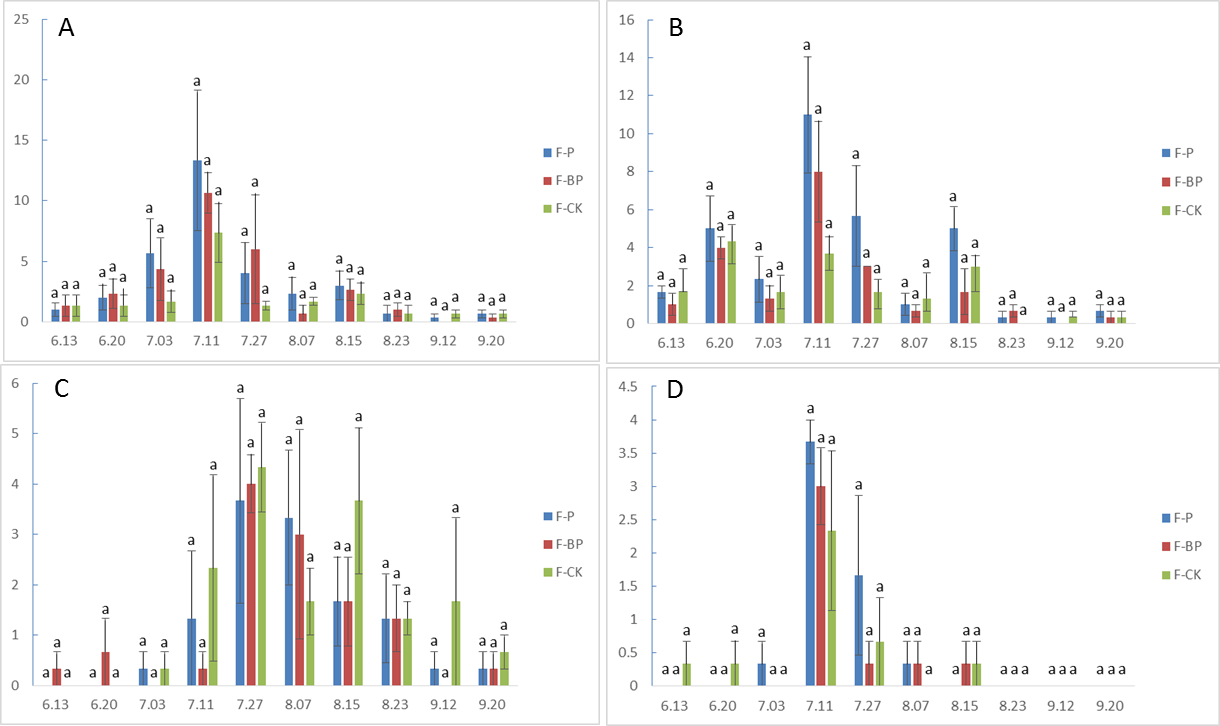
**

**Supplementary Figure 7.** Effects on arthropod community structure (Pitfall trap method). (A) *Teleogryllus infernalis*; (B) Earwig furficulidae; (**C**) opiliones; (**D)** *Carabidae sp.* F-CK: Non-Bt maize control; F-P: Cry1Ab/Gc maize spraying herbicide glufosinate; F-BP: Cry1Ab/Gc maize with no glufosinate application. The ordinate represents the average number of individuals in each community ± SD, and the abscissa represents survey time. Statistically significant differences according to one-way ANOVA (a: P>0.05).


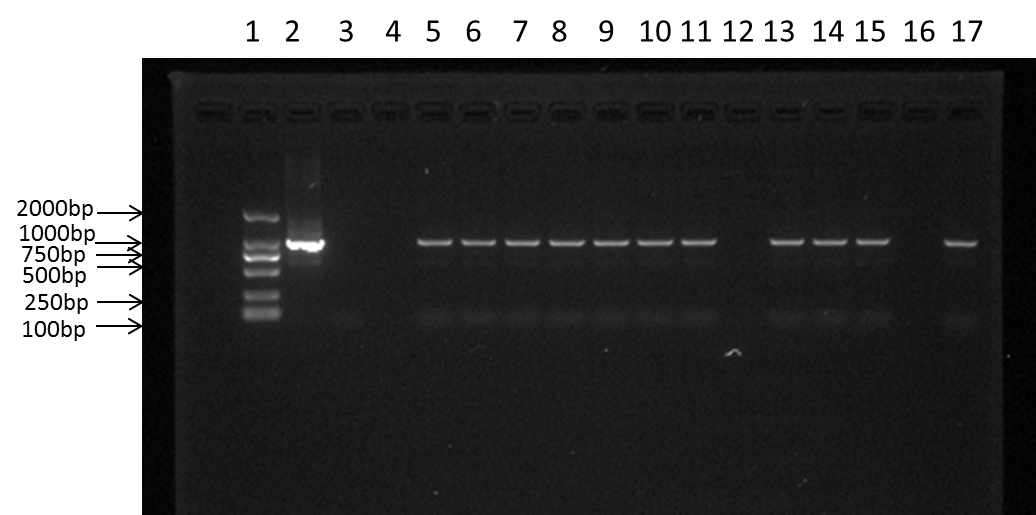


**Supplementary Figure 8.** PCR analysis of rice genomic DNA. 1: DL 2000 Marker; 2: Plasmid positive control; 3: Black control; 4: Negative Control; 5: Transgenic event C-1; 6: Transgenic event C-2; 7: Transgenic event C-3; 8-17: Other transgenic events


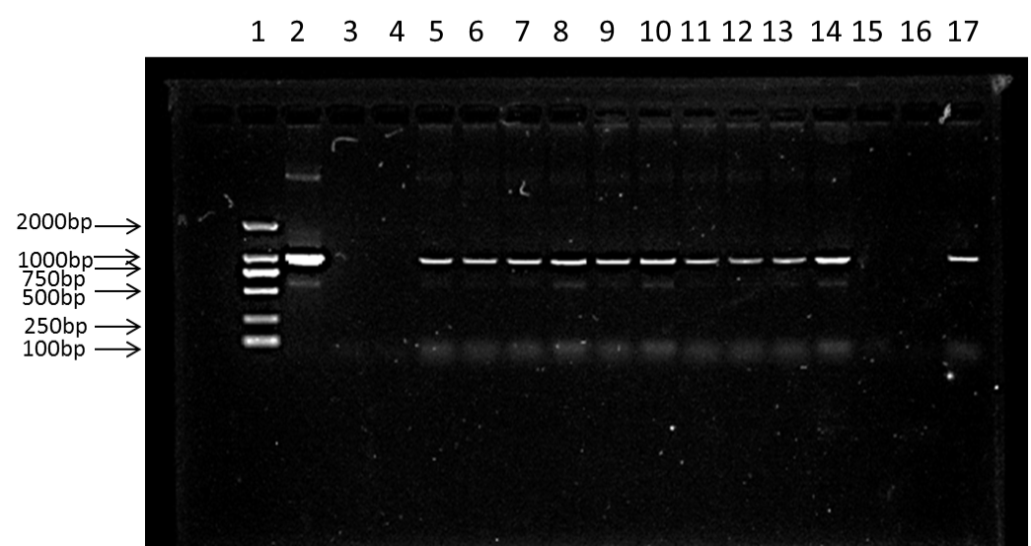


**Supplementary Figure 9.** PCR analysis of maize genomic DNA. 1: DL 2000 Marker; 2: Plasmid positive control; 3: Black control; 4: Negative Control; 5: Transgenic event HG-1; 6: Transgenic event HG-2; 7: Transgenic event HG-3; 8-17: Other transgenic events

**
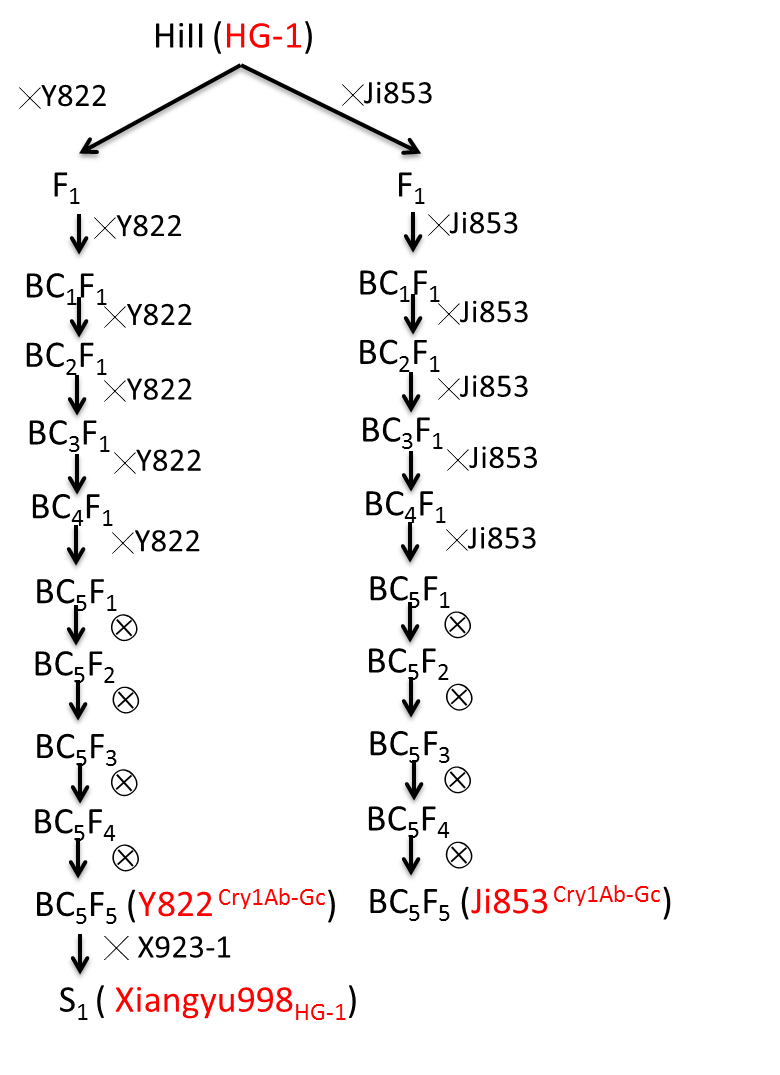
**

**Supplementary Figure 10.** Breeding flow of transgenic events. The genetically modified material used in the study is marked in red

**Supplementary Table 1.** Mortality rate of rice stem borer feeding on rice stems

| Plant line | No. of rice stem borers | Average mortality rate of rice stem borer (%) |
| --- | --- | --- |
| CK | 50 | 18.00±0.13 |
| *cry1Ab-Gc* OE | 50 | 100.00±0.00^**^ |
| *cry1Ab-Ac* OE | 50 | 96.00±0.00^**^ |
| *cry1Ab-Ia* OE | 50 | 96.00±0.00^**^ |

Notes: Means (±SE) within a column followed by different letters are significantly different (*t* -test, ***P*< 0.01).

**Supplementary Table 2.** Insect resistance of transgenic lines against Asian corn borer

| Maize lines | No. of plants | No. of channels | Length of tunnel (cm) | No. of live larvae |
| --- | --- | --- | --- | --- |
| CK | 12 | 2.92±0.62 | 4.75±1.23 | 0.75±0.25 |
| HG-1 | 14 | 0.36±0.17 ^**^ | 1.36±1.00 ^**^ | 0.00±0.00 ^**^ |
| HG-2 | 11 | 0.82±0.30 ^**^ | 0.91±0.67 ^**^ | 0.00±0.00 ^**^ |
| HG-3 | 15 | 0.80±0.20 ^**^ | 1.83±0.69 ^**^ | 0.13±0.13 ^**^ |

Notes: Means (±SE) within a column followed by different letters are significantly different (T test, ***P*< 0.01).

**Supplementary Table 3** Primer information

| Genes | Primer-F(5'-3') | Primer -R(5'-3') | PCR product length (bp) | vector |
| --- | --- | --- | --- | --- |
| *cry1Ab-1Gc* | TACAGATCATGCTGTACG | ATTCTAAGTTTTCCCCTC | 1085 | pET28a-cry1Ab-1Gc |
| *cry1Ab-1Ia* | ATTGTTAGCCTGTTCCCG | TATACACTTCATTGCCGC | 1075 | pET28a-cry1Ab-1Ia |
| *cry1Ab-1Ac* | TCTGACCCGCCTGATTGG | AGATGCTGCTGTTGCCCC | 1073 | pET28a-cry1Ab-1Ac |
| *cry1Ab-9Ca* | AACGTTGGGGCTTTGATG | CAGGCGATACTGTTGGGT | 1037 | pET28a-cry1Ab-9Ca |
| *cry1Ab-2Ba* | CGATGTGAGTGTGTTTGG | AGGCTGGTGTTATTGAAG | 1041 | pET28a-cry1Ab-2Ba |
| *cry1Ab-2Ae* | TGCGCGATGTGAGTGTGT | GTATAGCGGGCGGTGGTA | 1021 | pET28a-cry1Ab-2Ae |
| *cry1Ab-1Jb* | GTGCTGCGCGATGTGAGT | AGCGAAAGCGAATACGGT | 1075 | pET28a-cry1Ab-1Jb |
| *cry1Ab-Gc* | GGTACAATACAGGTCTTG | ACGGTCTATGGTCTTAGG | 1049 | pTF101.1 -cry1Ab*-*Gc |
| *cry1Ab-Ac* | GTGTTTGGGCAGAGATGG | TAGAGGGGAAGTGGATCG | 1045 | pTF101.1- cry1Ab*-*Ac |
| *cry1Ab-Ia* | AGATAGTCGGGATTGGAT | GTGCGGAATGTCTTGTAA | 1047 | pTF101.1- cry1Ab*-*Ia |

**Supplementary sequence 1:** Domain I and Domain II of Cry1Ab protein

MDNNPNINECIPYNCLSNPEVEVLGGERIETGYTPIDISLSLTQFLLSEFVPGAGFVLGLVDIIWGIFGPSQWDAFLVQIEQLINQRIEEFARNQAISRLEGLSNLYQIYAESFREWEADPTNPALREEMRIQFNDMNSALTTAIPLFAVQNYQVPLLSVYVQAANLHLSVLRDVSVFGQRWGFDAATINSRYNDLTRLIGNYTDHAVRWYNTGLERVWGPDSRDWIRYNQFRRELTLTVLDIVSLFPNYDSRTYPIRTVSQLTREIYTNPVLENFDGSFRGSAQGIEGSIRSPHLMDILNSITIYTDAHRGEYYWSGHQIMASPVGFSGPEFTFPLYGTMGNAAPQQRIVAQLGQGVYRTLSSTLYRRPFNIGINNQQLSVLDGTEFAYGTSSNLPSAVYRKSGTVDSLDEIPPQNNNVPPRQGFSHRLSHVSMFRSGFSNSSVSIIRAPMFSWIHRSAEFNNII

**Supplementary sequence 2:** Domain III of Cry1Ab protein

PSSQITQIPLTKSTNLGSGTSVVKGPGFTGGDILRRTSPGQISTLRVNITAPLSQRYRVRIRYASTTNLQFHTSIDGRPINQGNFSATMSSGSNLQSGSFRTVGFTTPFNFSNGSSVFTLSAHVFNSGNEVYIDRIEFVPAE

**Supplementary sequence 3:** Domain III of Cry1Ab-1Gc protein

ASDSGSITQLPMVKAHTLHAGATVVRGPGFTGGDILRRTTSGSFGDMRITNFSSSSSRYRVRIRYASTTDLQFFLNVGGTPVNVADFPKTIDRGENLEYGSFRTAGFTTPFSFVSSTNNFTLGVQSVSSGNEIFVDRIEFVPAD

**Supplementary sequence 4:** Domain III of Cry1Ab-1Ia protein

ASDSGSITQIPLVKAFNLSSGAAVVRGPGFTGGDILRRTNTGTFGDIRVNINPPFAQRYRVRIRYASTTDLQFHTSINGKAINQGNFSATMNRGEDLDYKTFRTVGFTTPFSFLDVQSTFTIGAWNFSSGNEVYIDRIEFVPVE

**Supplementary sequence 5:** Domain III of Cry1Ab-1Ac protein

ASDSGSITQIPAVKGNFLFNGSVISGPGFTGGDLVRLNSSGNNIQNRGYIEVPIHFPSTSTRYRVRVRYASVTPIHLNVNWGNSSIFSNTVPATATSLDNLQSSDFGYFESANAFTSSLGNIVGVRNFSGTAGVIIDRFEFIPVT

**Supplementary sequence 6:** Domain III of Cry1Ab-9Ca protein

PSSQITQLPLVKASAPVSGTTVLKGPGFTGGGILRRTTNGTFGTLRVTVNSPLTQQYRLRVRFASTGNFSIRVLRGGVSIGDVRLGSTMNRGQELTYESFFTREFTTTGPFNPPFTFTQAQEILTVNAEGVSTGGEYYIDRIEIVPVN

**Supplementary sequence 7:** Domain III of Cry1Ab-2Ba protein

PSSQITQIPLVKSFNLNSGTSVVSGPGFTGGDIIRTNVNGSVLSMGLNFNNTSLQRYRVRVRYAASQTMVLRVTVGGSTTFDQGFPSTMSANESLTSQSFRFAEFPVGISASGSQTAGISISNNAGRQTFHFDKIEFIPIT

**Supplementary sequence 8:** Domain III of Cry1Ab-2Ae protein

PSSQFTISPIHATQVNNQTRTFISEKFGNQGDSLRFEQSNTTARYTLRGNGNSYNLYLRVSSLGNSTIRVTINGRVYTASNVNTTTNNDGVNDNGARFLDINMGNVVASDNTNVPLDINVTFNSGTQFELMNIMFVPTN

**Supplementary sequence 9:** Domain III of Cry1Ab-1Jb protein

PSSQITQIPMVKAYNLHAGATVVRGPGFTGGDILRRTSNGMVVTLRVDASAVRNQRYRIRFRYAATSNFYFVVRRGNLGVNGREIMKTMSTGEELKSASFVLGEFITPFNFFENQVPLQIEIQSLSPGGEVYLDKIEFIPAD

**Supplementary sequence 10:** the *E coli* codon-optimized coding sequence of *cry1Ab-1Gc* gene

ATGGATAACAACCCGAACATTAACGAATGCATTCCGTATAACTGCCTGAGCAACCCGGAAGTGGAAGTGCTGGGTGGTGAACGTATTGAAACCGGCTATACCCCGATTGATATTTCGCTGAGCCTGACCCAGTTCCTGCTGAGCGAATTTGTACCGGGCGCCGGCTTTGTTCTGGGCCTGGTGGATATTATTTGGGGTATTTTTGGCCCGAGCCAGTGGGATGCGTTTCTGGTGCAGATTGAACAGCTGATTAACCAGCGCATTGAAGAATTTGCGCGCAATCAGGCAATTAGCCGTCTGGAAGGCCTGAGCAATCTGTATCAGATTTACGCCGAAAGCTTTCGTGAATGGGAAGCCGATCCGACCAACCCGGCGCTGCGCGAAGAAATGCGCATTCAGTTTAACGATATGAACAGCGCGCTGACCACCGCGATTCCGCTGTTCGCGGTGCAGAATTACCAGGTGCCGCTGCTGAGCGTGTATGTTCAGGCGGCGAACCTGCACCTGAGCGTGCTGCGCGATGTGAGTGTGTTTGGCCAACGTTGGGGCTTTGATGCCGCGACCATTAATAGCCGTTACAACGATCTGACCCGCCTGATTGGCAATTATACCGATCATGCGGTGCGTTGGTATAACACCGGCCTGGAGCGTGTTTGGGGTCCGGACAGCCGCGACTGGATTCGCTACAATCAGTTCCGCCGCGAACTGACCCTGACCGTGCTGGACATTGTTAGCCTGTTCCCGAACTATGATAGCCGTACCTATCCGATTCGCACCGTGAGCCAGCTGACCCGCGAAATTTACACCAATCCGGTGCTGGAAAACTTCGATGGTAGCTTTCGTGGCAGCGCCCAAGGCATTGAAGGCAGCATTCGCAGCCCGCATCTGATGGATATTCTGAATAGCATTACCATTTATACGGATGCGCACCGCGGTGAATACTATTGGAGCGGGCATCAGATTATGGCATCGCCGGTGGGCTTCAGCGGCCCGGAATTTACCTTTCCGCTGTACGGCACCATGGGTAATGCGGCGCCGCAGCAGCGCATTGTGGCGCAGCTGGGCCAGGGCGTGTACCGCACGCTGAGCAGCACCCTGTACCGCCGCCCGTTTAACATCGGTATTAATAATCAGCAGCTGTCAGTTCTGGATGGCACCGAATTTGCCTATGGCACCAGCAGCAATCTGCCGAGCGCGGTGTATCGTAAAAGTGGCACCGTGGATTCGCTGGATGAAATTCCGCCGCAGAATAACAATGTGCCGCCGCGCCAGGGCTTCAGCCACCGTCTGTCACATGTGAGCATGTTTCGTAGCGGCTTCTCGAACAGCAGCGTGAGCATTATTCGTGCCCCGATGTTTAGCTGGATTCATCGCAGCGCGGAATTTAATAACATTATT

GCGAGCGATAGCGGCAGCATTACCCAGCTGCCGATGGTGAAAGCGCATACCCTGCACGCCGGCGCCACCGTTGTGCGCGGCCCGGGCTTTACCGGCGGCGATATCCTGCGCCGTACCACCAGCGGCAGCTTTGGCGATATGCGCATTACCAACTTCAGCAGCTCCAGCAGCCGCTACCGCGTTCGCATTCGCTACGCCAGCACCACGGATCTGCAGTTTTTTCTGAACGTGGGCGGCACCCCGGTGAACGTGGCGGATTTTCCGAAAACCATTGATCGCGGCGAAAATCTGGAATACGGCAGCTTCCGCACCGCGGGCTTTACCACCCCGTTCAGCTTTGTGAGCAGCACCAATAATTTTACCCTGGGCGTCCAGAGCGTGTCAAGCGGCAATGAAATTTTTGTGGATCGCATTGAATTTGTTCCGGCGGATTAA

**Supplementary sequence 11:** the *E coli* codon-optimized coding sequence of *cry1Ab-1Ia* gene

ATGGATAACAACCCGAACATTAACGAATGCATTCCGTATAACTGCCTGAGCAACCCGGAAGTGGAAGTGCTGGGTGGTGAACGTATTGAAACCGGCTATACCCCGATTGATATTTCGCTGAGCCTGACCCAGTTCCTGCTGAGCGAATTTGTACCGGGCGCCGGCTTTGTTCTGGGCCTGGTGGATATTATTTGGGGTATTTTTGGCCCGAGCCAGTGGGATGCGTTTCTGGTGCAGATTGAACAGCTGATTAACCAGCGCATTGAAGAATTTGCGCGCAATCAGGCAATTAGCCGTCTGGAAGGCCTGAGCAATCTGTATCAGATTTACGCCGAAAGCTTTCGTGAATGGGAAGCCGATCCGACCAACCCGGCGCTGCGCGAAGAAATGCGCATTCAGTTTAACGATATGAACAGCGCGCTGACCACCGCGATTCCGCTGTTCGCGGTGCAGAATTACCAGGTGCCGCTGCTGAGCGTGTATGTTCAGGCGGCGAACCTGCACCTGAGCGTGCTGCGCGATGTGAGTGTGTTTGGCCAACGTTGGGGCTTTGATGCCGCGACCATTAATAGCCGTTACAACGATCTGACCCGCCTGATTGGCAATTATACCGATCATGCGGTGCGTTGGTATAACACCGGCCTGGAGCGTGTTTGGGGTCCGGACAGCCGCGACTGGATTCGCTACAATCAGTTCCGCCGCGAACTGACCCTGACCGTGCTGGACATTGTTAGCCTGTTCCCGAACTATGATAGCCGTACCTATCCGATTCGCACCGTGAGCCAGCTGACCCGCGAAATTTACACCAATCCGGTGCTGGAAAACTTCGATGGTAGCTTTCGTGGCAGCGCCCAAGGCATTGAAGGCAGCATTCGCAGCCCGCATCTGATGGATATTCTGAATAGCATTACCATTTATACGGATGCGCACCGCGGTGAATACTATTGGAGCGGGCATCAGATTATGGCATCGCCGGTGGGCTTCAGCGGCCCGGAATTTACCTTTCCGCTGTACGGCACCATGGGTAATGCGGCGCCGCAGCAGCGCATTGTGGCGCAGCTGGGCCAGGGCGTGTACCGCACGCTGAGCAGCACCCTGTACCGCCGCCCGTTTAACATCGGTATTAATAATCAGCAGCTGTCAGTTCTGGATGGCACCGAATTTGCCTATGGCACCAGCAGCAATCTGCCGAGCGCGGTGTATCGTAAAAGTGGCACCGTGGATTCGCTGGATGAAATTCCGCCGCAGAATAACAATGTGCCGCCGCGCCAGGGCTTCAGCCACCGTCTGTCACATGTGAGCATGTTTCGTAGCGGCTTCTCGAACAGCAGCGTGAGCATTATTCGTGCCCCGATGTTTAGCTGGATTCATCGCAGCGCGGAATTTAATAACATTATT

GCGAGCGATAGCGGTAGCATTACCCAGATCCCGCTGGTGAAAGCGTTTAACCTGTCTAGCGGCGCGGCGGTGGTTCGTGGCCCGGGCTTTACCGGCGGCGATATCCTGCGTCGTACCAACACCGGCACCTTCGGCGATATTCGTGTGAATATTAACCCGCCGTTTGCCCAGCGCTATCGTGTGCGCATCCGCTATGCGAGCACCACCGATCTGCAGTTTCATACCAGCATTAACGGCAAAGCGATTAACCAGGGCAATTTCTCTGCGACCATGAACCGCGGCGAAGATCTGGATTACAAAACCTTTCGCACCGTGGGCTTTACCACCCCGTTTTCGTTTCTGGATGTGCAGAGCACCTTTACCATTGGCGCGTGGAACTTTAGCAGCGGCAATGAAGTGTATATTGATCGCATTGAATTTGTGCCGGTGGAATAA

**Supplementary sequence 12:** the *E coli* codon-optimized coding sequence of *cry1Ab-1Ac* gene

ATGGATAACAACCCGAACATTAACGAATGCATTCCGTATAACTGCCTGAGCAACCCGGAAGTGGAAGTGCTGGGTGGTGAACGTATTGAAACCGGCTATACCCCGATTGATATTTCGCTGAGCCTGACCCAGTTCCTGCTGAGCGAATTTGTACCGGGCGCCGGCTTTGTTCTGGGCCTGGTGGATATTATTTGGGGTATTTTTGGCCCGAGCCAGTGGGATGCGTTTCTGGTGCAGATTGAACAGCTGATTAACCAGCGCATTGAAGAATTTGCGCGCAATCAGGCAATTAGCCGTCTGGAAGGCCTGAGCAATCTGTATCAGATTTACGCCGAAAGCTTTCGTGAATGGGAAGCCGATCCGACCAACCCGGCGCTGCGCGAAGAAATGCGCATTCAGTTTAACGATATGAACAGCGCGCTGACCACCGCGATTCCGCTGTTCGCGGTGCAGAATTACCAGGTGCCGCTGCTGAGCGTGTATGTTCAGGCGGCGAACCTGCACCTGAGCGTGCTGCGCGATGTGAGTGTGTTTGGCCAACGTTGGGGCTTTGATGCCGCGACCATTAATAGCCGTTACAACGATCTGACCCGCCTGATTGGCAATTATACCGATCATGCGGTGCGTTGGTATAACACCGGCCTGGAGCGTGTTTGGGGTCCGGACAGCCGCGACTGGATTCGCTACAATCAGTTCCGCCGCGAACTGACCCTGACCGTGCTGGACATTGTTAGCCTGTTCCCGAACTATGATAGCCGTACCTATCCGATTCGCACCGTGAGCCAGCTGACCCGCGAAATTTACACCAATCCGGTGCTGGAAAACTTCGATGGTAGCTTTCGTGGCAGCGCCCAAGGCATTGAAGGCAGCATTCGCAGCCCGCATCTGATGGATATTCTGAATAGCATTACCATTTATACGGATGCGCACCGCGGTGAATACTATTGGAGCGGGCATCAGATTATGGCATCGCCGGTGGGCTTCAGCGGCCCGGAATTTACCTTTCCGCTGTACGGCACCATGGGTAATGCGGCGCCGCAGCAGCGCATTGTGGCGCAGCTGGGCCAGGGCGTGTACCGCACGCTGAGCAGCACCCTGTACCGCCGCCCGTTTAACATCGGTATTAATAATCAGCAGCTGTCAGTTCTGGATGGCACCGAATTTGCCTATGGCACCAGCAGCAATCTGCCGAGCGCGGTGTATCGTAAAAGTGGCACCGTGGATTCGCTGGATGAAATTCCGCCGCAGAATAACAATGTGCCGCCGCGCCAGGGCTTCAGCCACCGTCTGTCACATGTGAGCATGTTTCGTAGCGGCTTCTCGAACAGCAGCGTGAGCATTATTCGTGCCCCGATGTTTAGCTGGATTCATCGCAGCGCGGAATTTAATAACATTATT

GCCAGCGATAGCGGCAGCATTACCCAGATTCCGGCCGTGAAAGGCAACTTCCTGTTTAACGGCAGCGTGATTAGCGGCCCGGGTTTTACCGGCGGCGATCTGGTGCGTCTGAACAGCAGCGGCAACAACATTCAGAATCGCGGCTATATTGAAGTACCGATTCACTTTCCGAGCACCAGCACCCGCTATCGCGTGCGCGTGCGCTATGCCAGCGTGACCCCGATTCACCTGAACGTGAATTGGGGCAACAGCAGCATCTTTAGTAATACCGTGCCGGCCACCGCGACCAGCCTGGATAACCTGCAGAGCAGCGATTTTGGCTACTTTGAAAGCGCGAACGCGTTCACCAGCAGCCTGGGCAACATTGTGGGTGTGCGTAACTTCAGCGGCACCGCCGGCGTGATTATTGATCGTTTTGAATTCATCCCGGTGACCTAA

**Supplementary sequence 13:** the *E coli* codon-optimized coding sequence of *cry1Ab-9Ca* gene

ATGGATAACAACCCGAACATTAACGAATGCATTCCGTATAACTGCCTGAGCAACCCGGAAGTGGAAGTGCTGGGTGGTGAACGTATTGAAACCGGCTATACCCCGATTGATATTTCGCTGAGCCTGACCCAGTTCCTGCTGAGCGAATTTGTACCGGGCGCCGGCTTTGTTCTGGGCCTGGTGGATATTATTTGGGGTATTTTTGGCCCGAGCCAGTGGGATGCGTTTCTGGTGCAGATTGAACAGCTGATTAACCAGCGCATTGAAGAATTTGCGCGCAATCAGGCAATTAGCCGTCTGGAAGGCCTGAGCAATCTGTATCAGATTTACGCCGAAAGCTTTCGTGAATGGGAAGCCGATCCGACCAACCCGGCGCTGCGCGAAGAAATGCGCATTCAGTTTAACGATATGAACAGCGCGCTGACCACCGCGATTCCGCTGTTCGCGGTGCAGAATTACCAGGTGCCGCTGCTGAGCGTGTATGTTCAGGCGGCGAACCTGCACCTGAGCGTGCTGCGCGATGTGAGTGTGTTTGGCCAACGTTGGGGCTTTGATGCCGCGACCATTAATAGCCGTTACAACGATCTGACCCGCCTGATTGGCAATTATACCGATCATGCGGTGCGTTGGTATAACACCGGCCTGGAGCGTGTTTGGGGTCCGGACAGCCGCGACTGGATTCGCTACAATCAGTTCCGCCGCGAACTGACCCTGACCGTGCTGGACATTGTTAGCCTGTTCCCGAACTATGATAGCCGTACCTATCCGATTCGCACCGTGAGCCAGCTGACCCGCGAAATTTACACCAATCCGGTGCTGGAAAACTTCGATGGTAGCTTTCGTGGCAGCGCCCAAGGCATTGAAGGCAGCATTCGCAGCCCGCATCTGATGGATATTCTGAATAGCATTACCATTTATACGGATGCGCACCGCGGTGAATACTATTGGAGCGGGCATCAGATTATGGCATCGCCGGTGGGCTTCAGCGGCCCGGAATTTACCTTTCCGCTGTACGGCACCATGGGTAATGCGGCGCCGCAGCAGCGCATTGTGGCGCAGCTGGGCCAGGGCGTGTACCGCACGCTGAGCAGCACCCTGTACCGCCGCCCGTTTAACATCGGTATTAATAATCAGCAGCTGTCAGTTCTGGATGGCACCGAATTTGCCTATGGCACCAGCAGCAATCTGCCGAGCGCGGTGTATCGTAAAAGTGGCACCGTGGATTCGCTGGATGAAATTCCGCCGCAGAATAACAATGTGCCGCCGCGCCAGGGCTTCAGCCACCGTCTGTCACATGTGAGCATGTTTCGTAGCGGCTTCTCGAACAGCAGCGTGAGCATTATTCGTGCCCCGATGTTTAGCTGGATTCATCGCAGCGCGGAATTTAATAACATTATT

CCGAGCAGCCAGATTACCCAGCTGCCGTTAGTGAAAGCCAGCGCACCGGTGAGCGGCACGACCGTGCTGAAAGGCCCGGGCTTTACCGGCGGCGGCATTCTGCGTCGCACGACCAACGGCACCTTTGGCACCCTGCGCGTCACCGTGAACAGCCCGCTGACCCAACAGTATCGCCTGCGCGTGCGTTTTGCGAGCACCGGCAACTTTTCAATTCGCGTGCTGCGCGGCGGCGTGAGCATTGGCGATGTGCGCCTGGGCTCAACCATGAATCGCGGCCAGGAACTGACCTATGAAAGCTTTTTTACCCGTGAATTTACCACCACCGGCCCGTTCAACCCGCCGTTTACCTTCACCCAGGCCCAGGAAATTCTGACCGTGAACGCGGAAGGCGTGAGCACCGGCGGCGAATACTACATTGATCGCATTGAAATTGTGCCGGTTAACTAA

**Supplementary sequence 14:** the *E coli* codon-optimized coding sequence of *cry1Ab-2Ba* gene

ATGGATAACAACCCGAACATTAACGAATGCATTCCGTATAACTGCCTGAGCAACCCGGAAGTGGAAGTGCTGGGTGGTGAACGTATTGAAACCGGCTATACCCCGATTGATATTTCGCTGAGCCTGACCCAGTTCCTGCTGAGCGAATTTGTACCGGGCGCCGGCTTTGTTCTGGGCCTGGTGGATATTATTTGGGGTATTTTTGGCCCGAGCCAGTGGGATGCGTTTCTGGTGCAGATTGAACAGCTGATTAACCAGCGCATTGAAGAATTTGCGCGCAATCAGGCAATTAGCCGTCTGGAAGGCCTGAGCAATCTGTATCAGATTTACGCCGAAAGCTTTCGTGAATGGGAAGCCGATCCGACCAACCCGGCGCTGCGCGAAGAAATGCGCATTCAGTTTAACGATATGAACAGCGCGCTGACCACCGCGATTCCGCTGTTCGCGGTGCAGAATTACCAGGTGCCGCTGCTGAGCGTGTATGTTCAGGCGGCGAACCTGCACCTGAGCGTGCTGCGCGATGTGAGTGTGTTTGGCCAACGTTGGGGCTTTGATGCCGCGACCATTAATAGCCGTTACAACGATCTGACCCGCCTGATTGGCAATTATACCGATCATGCGGTGCGTTGGTATAACACCGGCCTGGAGCGTGTTTGGGGTCCGGACAGCCGCGACTGGATTCGCTACAATCAGTTCCGCCGCGAACTGACCCTGACCGTGCTGGACATTGTTAGCCTGTTCCCGAACTATGATAGCCGTACCTATCCGATTCGCACCGTGAGCCAGCTGACCCGCGAAATTTACACCAATCCGGTGCTGGAAAACTTCGATGGTAGCTTTCGTGGCAGCGCCCAAGGCATTGAAGGCAGCATTCGCAGCCCGCATCTGATGGATATTCTGAATAGCATTACCATTTATACGGATGCGCACCGCGGTGAATACTATTGGAGCGGGCATCAGATTATGGCATCGCCGGTGGGCTTCAGCGGCCCGGAATTTACCTTTCCGCTGTACGGCACCATGGGTAATGCGGCGCCGCAGCAGCGCATTGTGGCGCAGCTGGGCCAGGGCGTGTACCGCACGCTGAGCAGCACCCTGTACCGCCGCCCGTTTAACATCGGTATTAATAATCAGCAGCTGTCAGTTCTGGATGGCACCGAATTTGCCTATGGCACCAGCAGCAATCTGCCGAGCGCGGTGTATCGTAAAAGTGGCACCGTGGATTCGCTGGATGAAATTCCGCCGCAGAATAACAATGTGCCGCCGCGCCAGGGCTTCAGCCACCGTCTGTCACATGTGAGCATGTTTCGTAGCGGCTTCTCGAACAGCAGCGTGAGCATTATTCGTGCCCCGATGTTTAGCTGGATTCATCGCAGCGCGGAATTTAATAACATTATT

CCGAGCAGCCAGATTACCCAGATTCCGCTGGTGAAAAGCTTTAATCTGAACAGCGGCACCAGCGTGGTGAGCGGTCCGGGCTTTACCGGCGGCGACATTATTCGCACGAACGTGAACGGCAGCGTGCTGAGCATGGGCCTGAACTTCAATAACACCAGCCTGCAACGCTACCGCGTGCGCGTGCGCTATGCGGCGAGCCAGACCATGGTGCTGCGCGTTACCGTGGGCGGTAGCACCACCTTCGATCAGGGCTTCCCGAGCACCATGAGCGCGAACGAATCACTGACCAGCCAGAGCTTTCGTTTTGCGGAGTTTCCGGTGGGCATTAGCGCCAGCGGCTCACAGACCGCCGGCATTAGCATTAGCAACAACGCGGGCCGCCAGACCTTTCACTTTGACAAAATTGAATTTATTCCGATCACCTAA

**Supplementary sequence 15:** the *E coli* codon-optimized coding sequence of *cry1Ab-2Ae* gene

ATGGATAACAACCCGAACATTAACGAATGCATTCCGTATAACTGCCTGAGCAACCCGGAAGTGGAAGTGCTGGGTGGTGAACGTATTGAAACCGGCTATACCCCGATTGATATTTCGCTGAGCCTGACCCAGTTCCTGCTGAGCGAATTTGTACCGGGCGCCGGCTTTGTTCTGGGCCTGGTGGATATTATTTGGGGTATTTTTGGCCCGAGCCAGTGGGATGCGTTTCTGGTGCAGATTGAACAGCTGATTAACCAGCGCATTGAAGAATTTGCGCGCAATCAGGCAATTAGCCGTCTGGAAGGCCTGAGCAATCTGTATCAGATTTACGCCGAAAGCTTTCGTGAATGGGAAGCCGATCCGACCAACCCGGCGCTGCGCGAAGAAATGCGCATTCAGTTTAACGATATGAACAGCGCGCTGACCACCGCGATTCCGCTGTTCGCGGTGCAGAATTACCAGGTGCCGCTGCTGAGCGTGTATGTTCAGGCGGCGAACCTGCACCTGAGCGTGCTGCGCGATGTGAGTGTGTTTGGCCAACGTTGGGGCTTTGATGCCGCGACCATTAATAGCCGTTACAACGATCTGACCCGCCTGATTGGCAATTATACCGATCATGCGGTGCGTTGGTATAACACCGGCCTGGAGCGTGTTTGGGGTCCGGACAGCCGCGACTGGATTCGCTACAATCAGTTCCGCCGCGAACTGACCCTGACCGTGCTGGACATTGTTAGCCTGTTCCCGAACTATGATAGCCGTACCTATCCGATTCGCACCGTGAGCCAGCTGACCCGCGAAATTTACACCAATCCGGTGCTGGAAAACTTCGATGGTAGCTTTCGTGGCAGCGCCCAAGGCATTGAAGGCAGCATTCGCAGCCCGCATCTGATGGATATTCTGAATAGCATTACCATTTATACGGATGCGCACCGCGGTGAATACTATTGGAGCGGGCATCAGATTATGGCATCGCCGGTGGGCTTCAGCGGCCCGGAATTTACCTTTCCGCTGTACGGCACCATGGGTAATGCGGCGCCGCAGCAGCGCATTGTGGCGCAGCTGGGCCAGGGCGTGTACCGCACGCTGAGCAGCACCCTGTACCGCCGCCCGTTTAACATCGGTATTAATAATCAGCAGCTGTCAGTTCTGGATGGCACCGAATTTGCCTATGGCACCAGCAGCAATCTGCCGAGCGCGGTGTATCGTAAAAGTGGCACCGTGGATTCGCTGGATGAAATTCCGCCGCAGAATAACAATGTGCCGCCGCGCCAGGGCTTCAGCCACCGTCTGTCACATGTGAGCATGTTTCGTAGCGGCTTCTCGAACAGCAGCGTGAGCATTATTCGTGCCCCGATGTTTAGCTGGATTCATCGCAGCGCGGAATTTAATAACATTATT

CCGAGCAGCCAGTTCACCATTAGCCCGATTCATGCGACCCAGGTGAACAATCAGACCCGCACCTTTATTAGCGAAAAATTTGGTAATCAAGGCGATAGCCTGCGTTTTGAACAGAGCAATACCACCGCCCGCTATACCCTGCGCGGCAATGGCAACAGCTATAACCTGTACCTGCGTGTGAGCAGCCTGGGCAACAGCACCATTCGTGTGACCATTAACGGCCGCGTGTATACCGCGAGCAACGTGAATACCACCACCAACAATGATGGCGTGAACGATAACGGTGCGCGCTTCCTGGATATTAACATGGGCAACGTCGTGGCGAGCGATAATACCAATGTGCCGCTGGATATTAATGTTACCTTTAATAGCGGCACCCAGTTTGAACTGATGAACATTATGTTTGTTCCGACCAACTGA

**Supplementary sequence 16:** the *E coli* codon-optimized coding sequence of *cry1Ab-1Jb* gene

ATGGATAACAACCCGAACATTAACGAATGCATTCCGTATAACTGCCTGAGCAACCCGGAAGTGGAAGTGCTGGGTGGTGAACGTATTGAAACCGGCTATACCCCGATTGATATTTCGCTGAGCCTGACCCAGTTCCTGCTGAGCGAATTTGTACCGGGCGCCGGCTTTGTTCTGGGCCTGGTGGATATTATTTGGGGTATTTTTGGCCCGAGCCAGTGGGATGCGTTTCTGGTGCAGATTGAACAGCTGATTAACCAGCGCATTGAAGAATTTGCGCGCAATCAGGCAATTAGCCGTCTGGAAGGCCTGAGCAATCTGTATCAGATTTACGCCGAAAGCTTTCGTGAATGGGAAGCCGATCCGACCAACCCGGCGCTGCGCGAAGAAATGCGCATTCAGTTTAACGATATGAACAGCGCGCTGACCACCGCGATTCCGCTGTTCGCGGTGCAGAATTACCAGGTGCCGCTGCTGAGCGTGTATGTTCAGGCGGCGAACCTGCACCTGAGCGTGCTGCGCGATGTGAGTGTGTTTGGCCAACGTTGGGGCTTTGATGCCGCGACCATTAATAGCCGTTACAACGATCTGACCCGCCTGATTGGCAATTATACCGATCATGCGGTGCGTTGGTATAACACCGGCCTGGAGCGTGTTTGGGGTCCGGACAGCCGCGACTGGATTCGCTACAATCAGTTCCGCCGCGAACTGACCCTGACCGTGCTGGACATTGTTAGCCTGTTCCCGAACTATGATAGCCGTACCTATCCGATTCGCACCGTGAGCCAGCTGACCCGCGAAATTTACACCAATCCGGTGCTGGAAAACTTCGATGGTAGCTTTCGTGGCAGCGCCCAAGGCATTGAAGGCAGCATTCGCAGCCCGCATCTGATGGATATTCTGAATAGCATTACCATTTATACGGATGCGCACCGCGGTGAATACTATTGGAGCGGGCATCAGATTATGGCATCGCCGGTGGGCTTCAGCGGCCCGGAATTTACCTTTCCGCTGTACGGCACCATGGGTAATGCGGCGCCGCAGCAGCGCATTGTGGCGCAGCTGGGCCAGGGCGTGTACCGCACGCTGAGCAGCACCCTGTACCGCCGCCCGTTTAACATCGGTATTAATAATCAGCAGCTGTCAGTTCTGGATGGCACCGAATTTGCCTATGGCACCAGCAGCAATCTGCCGAGCGCGGTGTATCGTAAAAGTGGCACCGTGGATTCGCTGGATGAAATTCCGCCGCAGAATAACAATGTGCCGCCGCGCCAGGGCTTCAGCCACCGTCTGTCACATGTGAGCATGTTTCGTAGCGGCTTCTCGAACAGCAGCGTGAGCATTATTCGTGCCCCGATGTTTAGCTGGATTCATCGCAGCGCGGAATTTAATAACATTATT

CCGAGCAGCCAGATTACCCAGATCCCGATGGTGAAAGCGTACAATCTGCATGCCGGCGCGACCGTGGTGCGCGGCCCGGGCTTTACCGGCGGCGATATCCTGCGCCGCACCAGCAACGGCATGGTGGTGACCCTGCGCGTGGATGCGAGCGCGGTGCGCAACCAGCGCTACCGTATTCGCTTTCGCTACGCCGCGACCAGCAACTTCTATTTTGTGGTGCGTCGCGGTAACCTGGGCGTGAACGGCCGCGAAATTATGAAAACGATGAGTACCGGCGAAGAACTGAAAAGCGCGAGCTTTGTGCTGGGCGAATTCATTACCCCGTTTAACTTTTTTGAAAACCAGGTGCCGCTGCAGATTGAAATTCAGAGCCTGAGCCCGGGCGGCGAAGTGTACCTGGATAAAATCGAATTCATTCCGGCGGATTAA

**Supplementary sequence 17:** the plant codon-optimized coding sequence of *cry1Ab-Gc* gene：

ATGGACAACAATCCGAACATAAACGAGTGCATTCCGTACAACTGCCTTTCTAACCCCGAGGTTGAGGTTCTTGGTGGTGAGCGTATTGAGACCGGCTACACCCCCATCGACATCAGCCTCTCACTCACTCAATTCTTGCTCTCCGAATTTGTGCCAGGTGCAGGCTTCGTGCTCGGCCTGGTGGATATTATCTGGGGGATTTTCGGGCCCTCGCAATGGGACGCGTTCCTCGTGCAGATCGAGCAACTCATCAATCAGCGCATCGAGGAGTTCGCGAGAAACCAAGCGATCTCTAGGTTGGAAGGCTTGTCAAATTTGTACCAGATCTACGCCGAGTCTTTCCGGGAGTGGGAAGCCGACCCCACCAATCCAGCGCTCAGGGAAGAAATGCGGATTCAGTTCAACGATATGAATAGCGCCCTGACTACGGCCATTCCTCTCTTCGCGGTCCAAAATTATCAGGTTCCACTTTTGTCCGTTTACGTCCAGGCAGCTAACTTGCACTTGTCTGTTCTCAGGGACGTGTCTGTGTTTGGGCAGAGATGGGGATTCGATGCCGCGACCATCAACTCACGCTACAACGACCTTACTCGCCTTATTGGCAACTACACTGATCACGCCGTCAGGTGGTACAATACAGGTCTTGAGCGGGTCTGGGGCCCAGATAGTCGGGATTGGATTCGCTACAATCAATTCAGGCGCGAGCTGACCCTGACGGTCCTCGACATTGTGAGTCTCTTTCCGAACTATGATTCCCGCACCTACCCGATTCGCACTGTGTCACAACTGACTAGGGAGATCTATACTAATCCCGTGCTGGAGAATTTCGACGGATCTTTTAGAGGTTCCGCCCAGGGGATTGAGGGCTCGATTAGGTCGCCACACCTGATGGATATACTCAACAGCATCACAATCTACACAGACGCTCACCGCGGAGAGTACTATTGGTCGGGTCACCAGATCATGGCATCGCCAGTGGGGTTCTCTGGACCGGAATTTACTTTTCCATTGTATGGGACAATGGGCAACGCCGCTCCACAGCAGAGAATCGTGGCGCAGCTCGGACAGGGTGTCTATCGGACCCTTAGCTCCACTCTTTATAGACGGCCGTTTAACATCGGCATCAATAATCAACAGCTCAGCGTTCTCGACGGAACCGAGTTTGCTTACGGAACGTCGTCCAATCTGCCTTCCGCTGTCTACCGGAAGTCCGGGACCGTGGACTCCTTGGACGAGATACCTCCTCAGAATAATAACGTGCCTCCTCGCCAGGGATTCAGTCATCGGCTTTCGCACGTGTCTATGTTCCGCAGCGGGTTCTCAAATTCCTCAGTGTCCATTATCCGCGCTCCTATGTTCTCCTGGATTCACCGTTCAGCCGAGTTCAATAATATCATCGCTTCCGATTCAGGATCCATTACGCAACTCCCAATGGTTAAAGCACACACTTTGCATGCTGGTGCCACGGTGGTTAGGGGTCCGGGTTTCACAGGGGGAGACATCCTGCGGCGCACCACCTCGGGGAGCTTCGGCGACATGAGGATCACAAATTTTTCGTCCAGTAGTAGCAGGTACCGGGTCCGCATTAGATACGCTTCAACAACGGATCTTCAGTTCTTCCTGAACGTCGGCGGGACTCCGGTCAACGTGGCGGATTTTCCTAAGACCATAGACCGTGGAGAGAATCTTGAATATGGCAGCTTCCGCACCGCCGGCTTCACCACTCCGTTCTCCTTCGTCTCCTCTACTAACAATTTTACGCTCGGCGTGCAGTCAGTGTCTTCCGGCAACGAGATCTTCGTTGATAGAATCGAGTTTGTGCCCGCGGACTGA

**Supplementary sequence 18:** the plant codon-optimized coding sequence of *cry1Ab-Ac* gene：

ATGGACAACAATCCGAACATAAACGAGTGCATTCCGTACAACTGCCTTTCTAACCCCGAGGTTGAGGTTCTTGGTGGTGAGCGTATTGAGACCGGCTACACCCCCATCGACATCAGCCTCTCACTCACTCAATTCTTGCTCTCCGAATTTGTGCCAGGTGCAGGCTTCGTGCTCGGCCTGGTGGATATTATCTGGGGGATTTTCGGGCCCTCGCAATGGGACGCGTTCCTCGTGCAGATCGAGCAACTCATCAATCAGCGCATCGAGGAGTTCGCGAGAAACCAAGCGATCTCTAGGTTGGAAGGCTTGTCAAATTTGTACCAGATCTACGCCGAGTCTTTCCGGGAGTGGGAAGCCGACCCCACCAATCCAGCGCTCAGGGAAGAAATGCGGATTCAGTTCAACGATATGAATAGCGCCCTGACTACGGCCATTCCTCTCTTCGCGGTCCAAAATTATCAGGTTCCACTTTTGTCCGTTTACGTCCAGGCAGCTAACTTGCACTTGTCTGTTCTCAGGGACGTGTCTGTGTTTGGGCAGAGATGGGGATTCGATGCCGCGACCATCAACTCACGCTACAACGACCTTACTCGCCTTATTGGCAACTACACTGATCACGCCGTCAGGTGGTACAATACAGGTCTTGAGCGGGTCTGGGGCCCAGATAGTCGGGATTGGATTCGCTACAATCAATTCAGGCGCGAGCTGACCCTGACGGTCCTCGACATTGTGAGTCTCTTTCCGAACTATGATTCCCGCACCTACCCGATTCGCACTGTGTCACAACTGACTAGGGAGATCTATACTAATCCCGTGCTGGAGAATTTCGACGGATCTTTTAGAGGTTCCGCCCAGGGGATTGAGGGCTCGATTAGGTCGCCACACCTGATGGATATACTCAACAGCATCACAATCTACACAGACGCTCACCGCGGAGAGTACTATTGGTCGGGTCACCAGATCATGGCATCGCCAGTGGGGTTCTCTGGACCGGAATTTACTTTTCCATTGTATGGGACAATGGGCAACGCCGCTCCACAGCAGAGAATCGTGGCGCAGCTCGGACAGGGTGTCTATCGGACCCTTAGCTCCACTCTTTATAGACGGCCGTTTAACATCGGCATCAATAATCAACAGCTCAGCGTTCTCGACGGAACCGAGTTTGCTTACGGAACGTCGTCCAATCTGCCTTCCGCTGTCTACCGGAAGTCCGGGACCGTGGACTCCTTGGACGAGATACCTCCTCAGAATAATAACGTGCCTCCTCGCCAGGGATTCAGTCATCGGCTTTCGCACGTGTCTATGTTCCGCAGCGGGTTCTCAAATTCCTCAGTGTCCATTATCCGCGCTCCTATGTTCTCCTGGATTCACCGTTCAGCCGAGTTCAATAATATCATCGCTTCCGATTCAGGATCCATCACACAGATCCCGGCGGTGAAGGGAAACTTCCTTTTCAATGGCTCGGTGATTTCCGGACCGGGGTTTACCGGTGGCGATCTCGTGAGACTGAACTCGTCAGGGAACAATATCCAAAATAGGGGCTACATTGAGGTCCCGATCCACTTCCCCTCTACATCGACGAGATATCGTGTTCGGGTGAGATACGCATCGGTCACGCCAATTCATCTGAACGTGAACTGGGGCAACTCGTCCATATTCTCTAACACAGTGCCCGCGACAGCTACTAGCCTGGACAATCTTCAATCTAGCGATTTCGGTTACTTCGAGTCAGCCAATGCCTTCACTAGCAGTCTCGGCAACATAGTTGGCGTCCGCAACTTCAGCGGCACTGCTGGTGTGATCATCGACAGGTTCGAGTTCATACCGGTTACTTGA

**Supplementary sequence 19:** the plant codon-optimized coding sequence of *cry1Ab-Ia* gene：

ATGGACAACAATCCGAACATAAACGAGTGCATTCCGTACAACTGCCTTTCTAACCCCGAGGTTGAGGTTCTTGGTGGTGAGCGTATTGAGACCGGCTACACCCCCATCGACATCAGCCTCTCACTCACTCAATTCTTGCTCTCCGAATTTGTGCCAGGTGCAGGCTTCGTGCTCGGCCTGGTGGATATTATCTGGGGGATTTTCGGGCCCTCGCAATGGGACGCGTTCCTCGTGCAGATCGAGCAACTCATCAATCAGCGCATCGAGGAGTTCGCGAGAAACCAAGCGATCTCTAGGTTGGAAGGCTTGTCAAATTTGTACCAGATCTACGCCGAGTCTTTCCGGGAGTGGGAAGCCGACCCCACCAATCCAGCGCTCAGGGAAGAAATGCGGATTCAGTTCAACGATATGAATAGCGCCCTGACTACGGCCATTCCTCTCTTCGCGGTCCAAAATTATCAGGTTCCACTTTTGTCCGTTTACGTCCAGGCAGCTAACTTGCACTTGTCTGTTCTCAGGGACGTGTCTGTGTTTGGGCAGAGATGGGGATTCGATGCCGCGACCATCAACTCACGCTACAACGACCTTACTCGCCTTATTGGCAACTACACTGATCACGCCGTCAGGTGGTACAATACAGGTCTTGAGCGGGTCTGGGGCCCAGATAGTCGGGATTGGATTCGCTACAATCAATTCAGGCGCGAGCTGACCCTGACGGTCCTCGACATTGTGAGTCTCTTTCCGAACTATGATTCCCGCACCTACCCGATTCGCACTGTGTCACAACTGACTAGGGAGATCTATACTAATCCCGTGCTGGAGAATTTCGACGGATCTTTTAGAGGTTCCGCCCAGGGGATTGAGGGCTCGATTAGGTCGCCACACCTGATGGATATACTCAACAGCATCACAATCTACACAGACGCTCACCGCGGAGAGTACTATTGGTCGGGTCACCAGATCATGGCATCGCCAGTGGGGTTCTCTGGACCGGAATTTACTTTTCCATTGTATGGGACAATGGGCAACGCCGCTCCACAGCAGAGAATCGTGGCGCAGCTCGGACAGGGTGTCTATCGGACCCTTAGCTCCACTCTTTATAGACGGCCGTTTAACATCGGCATCAATAATCAACAGCTCAGCGTTCTCGACGGAACCGAGTTTGCTTACGGAACGTCGTCCAATCTGCCTTCCGCTGTCTACCGGAAGTCCGGGACCGTGGACTCCTTGGACGAGATACCTCCTCAGAATAATAACGTGCCTCCTCGCCAGGGATTCAGTCATCGGCTTTCGCACGTGTCTATGTTCCGCAGCGGGTTCTCAAATTCCTCAGTGTCCATTATCCGCGCTCCTATGTTCTCCTGGATTCACCGTTCAGCCGAGTTCAATAATATCATCGCTTCCGATTCAGGATCCATAACACAGATCCCGCTGGTGAAAGCATTTAATCTTTCTAGCGGAGCTGCCGTTGTGAGGGGTCCAGGATTCACGGGCGGTGATATTTTGCGCCGGACCAACACCGGCACGTTCGGCGATATAAGGGTGAACATTAATCCTCCCTTTGCTCAAAGGTACCGTGTTCGCATCCGGTATGCCTCCACTACCGACCTCCAGTTCCACACCTCTATCAACGGTAAGGCGATCAACCAGGGCAATTTCTCAGCCACCATGAACAGAGGGGAGGACCTCGATTACAAGACATTCCGCACTGTCGGCTTCACGACCCCGTTTTCCTTCCTGGACGTTCAATCGACATTCACTATTGGGGCGTGGAATTTCAGCAGTGGGAACGAGGTGTACATCGACAGAATCGAGTTTGTCCCGGTCGAATGA
